# Vicarious Trial-and-Error Is Enhanced During Deliberation in Human Virtual Navigation in a Translational Foraging Task

**DOI:** 10.3389/fnbeh.2021.586159

**Published:** 2021-04-12

**Authors:** Thach Huynh, Keanan Alstatt, Samantha V. Abram, Neil Schmitzer-Torbert

**Affiliations:** ^1^Department of Psychology, Wabash College, Crawfordsville, IN, United States; ^2^Sierra Pacific Mental Illness Research Education and Clinical Centers, San Francisco Veterans Affairs Medical Center, Veterans Affairs San Francisco Healthcare System, University of California, San Francisco, San Francisco, CA, United States; ^3^Department of Psychiatry, University of California, San Francisco, San Francisco, CA, United States; ^4^Mental Health Service, Veterans Affairs San Francisco Healthcare System, San Francisco, CA, United States

**Keywords:** vicarious-trial-and-error, sunk-cost bias, deliberation (VTE), smoking, obesity

## Abstract

Foraging tasks provide valuable insights into decision-making as animals decide how to allocate limited resources (such as time). In rodents, vicarious trial-and-error (back and forth movements), or VTE, is an important behavioral measure of deliberation which is enhanced early in learning and when animals are presented with difficult decisions. Using new translational versions of a rodent foraging task (the “Movie Row” and “Candy Row”), humans navigated a virtual maze presented on standard computers to obtain rewards (either short videos or candy) offered after a variable delay. Decision latencies were longer when participants were presented with difficult offers, overrode their preferences, and when they accepted an offer after rejecting a previous offer. In these situations, humans showed VTE-like behavior, where they were more likely to pause and/or reorient one or more times before making a decision. Behavior on these tasks replicated previous results from the rodent foraging task (“Restaurant Row”) and a human version lacking a navigation component (“Web-Surf”) and revealed some species differences. Compared to survey measures of delay-discounting, willingness to wait for rewards in the foraging task was not related to willingness to wait for hypothetical rewards. And, smoking status (use of cigarettes or e-cigarettes) was associated with stronger discounting of hypothetical future rewards, but was not well-related to performance on the foraging tasks. In contrast, individuals with overweight or obese BMI (≥25) did not show stronger delay-discounting, but individuals with BMI ≥ 25, and especially females, showed reduced sensitivity to sunk-costs (where their decisions were less sensitive to irrecoverable investments of effort) and less deliberation when presented with difficult offers. These data indicate that VTE is a behavioral index of deliberation in humans, and further support the Movie and Candy Row as translational tools to study decision-making in humans with the potential to provide novel insights about decision-making that are relevant to public health.

## Introduction

Decision-making is a key capability, and in some sense a fundamental goal of the nervous system. Rather than a unitary construct, decisions rely on differentiable brain systems, each with a separate computational goal (Redish, 2013). Disadvantageous behaviors can be conceptualized as failures in one or more decision-making systems, and the ability to characterize different facets of the decision-making process is a critical part of the effort to understand normal and pathological decisions. In studies of deliberation, where one must search and evaluate options available in the current environment, vicarious trial-and-error (VTE, pause-and-look behavior) in rodents has been identified as an important behavioral index of deliberation [as reviewed in Redish (2016)]. For example, in the Restaurant Row task, rodents forage on a square track, navigating between sites that deliver different flavors of food rewards that are available after a variable delay. Rats and mice show increased VTE when presented with difficult offers (near their stay/skip thresholds for a reward), and in mice VTE is associated with better decisions, correlating with the probability of skipping a low-value offer (Steiner and Redish, 2014; Sweis et al., 2018b). Performance on the task has been used to study the differential impact of addictive drug exposure on decision-making. For example, long-term withdrawal from cocaine and heroin in mice produces dissociable effects on Restaurant Row performance (Sweis et al., 2018a): cocaine-abstinent mice show changes in deliberation, assessed by levels of VTE, while morphine-abstinent mice show a reduced willingness to quit after initially accepting an offer.

In humans, less evidence exists linking VTE-like measures to deliberation. Several studies have indicated that eye movements in human and non-human primates shares properties with VTE, with returns of fixation to previously sampled objects associated with better future memory (Voss et al., 2011), and better performance during difficult perceptual discriminations (Voss and Cohen, 2017). A study by Santos-Pata and Verschure (2018) found that VTE-like head movements were enhanced early in learning on a virtual maze, and were elevated specifically at an early (high-cost) decision point, similar to VTE behaviors in rats trained on a multiple T-maze (van der Meer et al., 2010). These results are encouraging, but whether VTE is a shared behavioral index of deliberation across species is not known.

A translational human version of Restaurant Row, called the Web-Surf task (Abram et al., 2016), uses short (4-s) video clips from different categories (kittens, bike accidents, landscapes and dancing) as rewards. Rather than navigating physically between reward sites, humans transition between different video galleries by repeatedly pressing a button. Abram et al. (2019a) have shown that decision latencies are elevated for difficult decisions on the Web-Surf task, similar to findings in rodents and consistent with the proposal that deliberation is enhanced for difficult offers. However, the original Web-Surf task did not allow for the assessment of VTE-like behaviors (i.e., physical pause and reorient patterns).

Beyond deliberation, behavior on the Web-Surf task converges with several important findings from Restaurant Row, and supports the use of these tasks to study decision-making processes shared across rodents and humans. For example, Sweis et al. (2018a) found that both humans and rodents demonstrated a sunk-cost effect on these tasks: after initially accepting an offer, the probability of completing the remainder of a delay increased as a function of the amount of time already invested in each species.

Foraging tasks (in which animals must allocate limited time or effort to obtain resources/rewards) can offer complementary insights into decision-making, compared to standard measures, such as forced-choice delay-discounting tasks (Stephens, 2008). In Restaurant Row and Web-Surf task, rewards of different types (i.e., flavor of food pellet, category of video) are available after a variable delay, and decisions to accept or reject offers are related to individual differences in one's willingness to wait for rewards of varying subjective quality. Performance on these tasks is altered in cocaine- and morphine-abstinent mice, while humans who score high on measures of addiction vulnerability show less behavior changes after risky losses on a variant of the Web-Surf task (Abram et al., 2019b). These results demonstrate that the willingness to wait for rewards may be related to drug exposure and addiction vulnerability, similar to research that has linked high levels of impulsivity to a range of negative outcomes.

Relative to foraging tasks, delay-discounting tasks typically assess impulsivity by presenting a forced-choice between real or hypothetical rewards of different magnitude available at different delays (Odum, 2011). Delay-discounting performance then reflects the degree to which rewards available in the future lose value compared to rewards available in the present. Discounting rates are elevated among individuals who smoke, use drugs, have problematic gambling, or are obese (MacKillop et al., 2011; Emery and Levine, 2017). Steep discounting of future rewards may represent a general risk factor that predisposes individuals to risky behaviors (like drug use), and/or discounting rates may be increased through experiences such as exposure to drugs or engaging in risky behaviors (Naude et al., 2015).

While discounting of future rewards has been implicated in a range of outcomes involving disadvantageous behaviors, decision-making involves a range of systems (Redish, 2013), and a more complete understanding of any particular condition requires assessment of a wider range of decision-making processes. For example, while both smokers and problematic gamblers show stronger discounting of future rewards, performance during foraging tasks shows a dissociation between these groups, with smokers showing a reduction in exploratory choices (in a multi-armed bandit task, Addicott et al., 2013), and gamblers showing an increase in exploratory choices (Addicott et al., 2015). Frequent gambling was also associated with increased exploratory choices in a 4-armed bandit task (where reward probabilities shifted for each of four options across a session), and with early, suboptimal, exits in a patchy foraging task (where reward available in each “patch” decreased as a function of the dwell time, and optimal behavior depends on the amount of reward remaining, and the time required to “travel” to the next patch), further indicating that gamblers showed increased exploration and reduced exploitation (Addicott et al., 2015). Thus, foraging tasks may be sensitive to different decision-making deficits than captured by typical delay discounting tasks.

To determine if VTE-like behaviors are associated with deliberation in human virtual navigation, we developed two virtual navigation versions of Restaurant Row (here referred to as the *Movie Row* and *Candy Row*). The tasks were presented on standard desktop and laptop computers, and required participants to navigate on a square track, similar to that used in the rodent Restaurant Row. At reward sites located on each corner of the track, participants were offered short video clips, taken from the Web-Surf task (Movie Row), or candy/snacks (Candy Row), which were available after a variable delay. VTE measures were assessed during the window from when an offer was presented until participants made a decision (to accept or reject the offer).

We predicted that when participants were presented with difficult offers (for delays close to a person's stay/skip threshold), the latency to make a decision would be elevated, and participants would show enhanced VTE (making one or more corrections before committing to their final choice). To quantify VTE, we focused on behavior observed within an offer zone, where participants made their decisions to accept or reject an offer for a specific reward. The primary outcome measures were decision latency (time from when the offer is presented until participants commit to accept or reject the offer by leaving the offer zone), the total amount of rotation (analogous to the rodent measure of VTE, the integrated absolute angular change in the orientation of head motion, Papale et al., 2012), reversals in rotation direction, total distance traveled, and cumulative time spent paused in the offer zone. We further expected that behavior on the Movie Row and Candy Row task would replicate the published behavioral findings from the Web-Surf task and Restaurant Row related to measures of regret (Steiner and Redish, 2014; Sweis et al., 2018c), sunk-costs (Sweis et al., 2018a), overriding reward preferences (Sweis et al., 2018b, see Supplementary Material), and sequences of choices (Abram et al., 2019a).

We also conducted a set of exploratory analyses, to determine the relationship between measures of delay discounting for money (the Monetary Choice Questionnaire, MCQ, Kirby et al., 1999) and food (the Food Choice Questionnaire, FCQ, Hendrickson et al., 2015) to behavioral measures derived from the Movie and Candy Row tasks. We predicted that discounting rates would be related across the two delay-discounting surveys, and that willingness to wait for rewards would be related across the two navigation tasks. Based on previous work with a version of the Web-Surf task involving risk (Abram et al., 2019b), we predicted that discounting rates would not be related to performance on the foraging tasks. From a subset of participants, information about smoking status and BMI were available from screening surveys. A final set of analyses were conducted to replicate previous reports that smoking and obesity are associated with stronger delay-discounting, and to explore the relationship between smoking status and obesity to behavior on the Movie and Candy Row tasks.

## Method

### Participants

Undergraduates (male, *n* = 144), workers from Amazon's Mechanical Turk service (mTurk, *n* = 147, 80 female/66 male/1 non-binary), and members of the local community (*n* = 34, 19 female/15 male) participated in the study. Race and ethnicity information were collected from some participants (*n* = 243), and most participants identified themselves as White (80.2%), with 4.9% identifying as African-American/Black, 7.4% as Asian, 4.5% as Hispanic or Latino, and the remainder selecting another option. Details on participant recruitment and compensation are described in the Supplementary Material. Participants provided informed consent before participating, and all procedures were approved by the Wabash College Institutional Review Board.

### Materials

#### Movie Row Task

The Movie Row was modeled on the rodent Restaurant Row (Steiner and Redish, 2014; Sweis et al., 2018a), and the human Web-Surf task (Abram et al., 2016). The computer task was implemented using the Unity 3D game engine (http://unity3d.com), embedded in a web page. Data collected by the task were saved in a MySQL database, submitted using custom PHP scripts.

**Figure 1:**
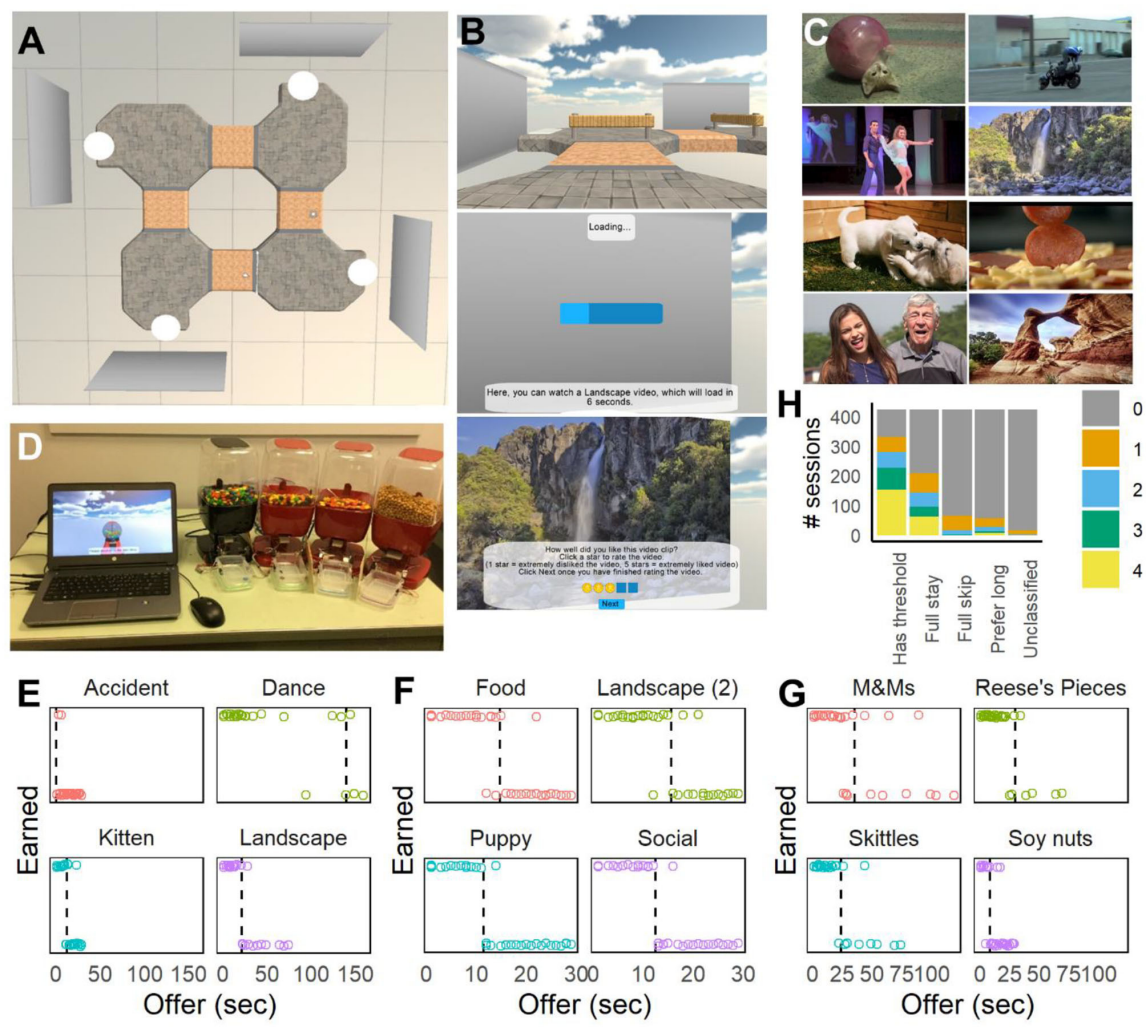
Virtual navigation tasks and choice behavior. **(A)** Overhead view of the Movie Row track used after Version 1. Four movie screens were used, each displaying a different type of video clip. **(B)** First-person screenshots taken during the task, showing navigation between offer zones (*top*), loading bar as the participant waits for an offer (*middle*), and showing the post-video rating after a video clip is played (*bottom*). **(C)** Examples of movies used in the Movie Row task from the Web-Surf task (*top*: kittens, accidents, dancing, and landscapes) and a second set (*bottom*: puppies, food, social, landscapes). **(D)** Setup for the Candy Row task and food dispensers. Movie screens in the task were replaced with virtual candy (gumball) dispensers. **(E–G)** Examples of choice behavior for one participant (DDR035) tested on the Movie Row task with the original **(E)** and new **(F)** videos, and on the Candy Row task **(G)**. Sessions from **(E,G)** were done in the lab, and the session from **(F)** was done by the participant online. **(H)** Distribution of the types of responding (number of delay thresholds, full-stay or full-skip behavior and preferences for long delays) across the sample.

Participants used standard desktop or laptop computers to navigate a virtual track to visit four reward zones sequentially, which each contained a movie screen (Figures 1A–C). Upon entering a reward zone, participants were presented with options to view short 4-s video clips from several categories (e.g., kittens, bike accidents, dancing, and scenes of landscapes: movies were taken from Abram et al., 2016, available after a variable delay. The movie type assigned to the screen in each reward zone was randomly determined for each participant. To accept the offer, they were required to step onto a white platform, and look at the movie screen. This would initiate the delay to receive a reward, which was signaled by a progress bar on the screen (Figure 1B). If they looked away, the delay would pause, hiding the progress bar, and would resume if they looked back at the screen. Participants could also skip movies entirely or quit during the delay [choice options were thus comparable to those in Sweis et al. (2018c)]. Each video clip was accompanied by audio and headphones were provided for participants tested in-person in groups in a computer lab. Movies were randomly selected from the set of those available for each category, and used without replacement. After each video clip, participants rated their enjoyment of the video (1 star = did not like the video, 5 stars = extremely liked the video). After completing the task, participants completed a post-task survey asking them to rank the videos from 1 = favorite to 4 = least favorite.

The task began with a practice phase, to introduce participants to the general task structure, and to the process of accepting and skipping offers. After completing the practice phase, participants completed a test phase. Four versions of the task were tested to refine the task for particular undergraduate research projects (testing the impact of glucose consumption, dieting/obesity, and smoking on decision-making). Details on the samples recruited for these studies and links to the current version of Movie Row are provided in the Supplementary Material.

##### Version 1

Participants navigated in a virtual environment, on a rounded square track. Four movie screens were positioned on the corners of the track, each of which played a different type of movie from the original Web-Surf task. Examples of each movie type are shown in Figure 1C, top. Participants entered an offer zone as they approached the screen (visible as a line on the track, though not explicitly described to the participant), where they were presented with the offer (the type of video, and the delay).

The task continued until a participant had received an offer for each delay for each movie type, or until 40 min in the main task had elapsed. Most finished much faster (about 30 min), unless they watched the majority of the movies. Delays (3–29 s) were randomly selected for each type, also without replacement. In the practice phase, participants were allowed to choose and skip movies until they had watched two videos of the same type (requiring at least one full circuit of the track).

##### Version 2

The practice phase was revised so that participants were required to skip and watch one video of each type (the order randomized for each movie screen). If participants accepted an offer that was within 5 s of the maximum delay, the maximum delay for that movie type was increased by 5 s (in order to better estimate delay thresholds when participants were willing to wait longer than 29 s for some video types). The track was revised as well (see Figure 1A) to refine the offer zone, requiring participants to turn ~90-degrees to their left to accept a video, and to turn 90-degrees to their right to skip a video and continue to the next video category. Along with video ratings, the post-task survey asked about their ability to hear the audio in the video clips, and asked participants to describe how they selected which videos to watch and skip.

##### Version 3

To assess reaction time, a gate was placed at the entrance to each offer zone. As participants arrived, their avatar was frozen until the gate lowered (over a window of 1 s) and the offer was presented. Reaction time was measured from the time the avatar was unfrozen until the participant started moving.

##### Version 4

While participants typically accepted offers with short delays, and skipped offers with long delays, occasionally participants demonstrated the opposite pattern and were more willing to wait for long delays. This behavior was more common in online samples, and occasionally was associated with reports by participants that they preferred long delays so that they could switch to another task during the delay. In an attempt to decrease this behavior, a fixation task was implemented during the delay, where two-digit numbers (between 10 and 99) were presented at random intervals during the delay. Once the delay was complete, participants were presented with four different two-digit numbers as options on the screen, and required to select the most recently presented number. After correct choices, the movie would play immediately. After incorrect responses, the message “Incorrect” was presented, and a 3 s time-out was implemented before the movie was started.

To facilitate the process of accepting an offer, the platform location was also expanded (to encompass the majority of the area outside of the offer zone), and a wider range of viewing angles (to the screen) were allowed to start the delay timer. With these changes, the delays would often start as soon as participants left the offer zone. However, this allowed participants to complete the delay from positions where the movie screen was only partially visible, or visible at an angle. So, videos were displayed immediately in front of the participant on a screen that was visible only when rewards were presented (rather than on the virtual screen used in earlier versions).

In the test phase, participants received offers with 1-s delays for the first three visits to each reward site, to provide participants with more experience with each reward before longer delays were implemented, and to better determine if participants who skipped the majority of offers for a video type would be willing to wait for even very short delays. If participants accepted an offer that was within the five longest delays available, the maximum delay was increased by 50 seconds (in ten increments of 5 seconds). For most participants, the maximum possible delay was fixed at 180 seconds. For version 4, some participants (*n* = 44) were tested with an alternate set of movies, including scenes of food, puppies, social interactions and landscapes (Figure 1C, bottom), provided by the laboratory of Aoife O'Donovan, and were selected from YouTube videos that were high quality (>1080 HD) and relevant to the category. Any text visible in the videos was removed by cropping.

#### Candy Row Task

The Movie Row task was adapted to deliver physical rewards (candy and snacks) using four commercial motion activated candy dispensers (Motion Activated Snack Dispenser, discontinued, Sharper Image) which delivered one of four rewards: M&Ms, Skittles, Reese's Pieces, or soy nuts/pepitas/other (see Figure 1D). The program was presented on a laptop computer, in full screen mode, rather than in a web page. Participants were tested individually either alone in a small room or in a classroom under the supervision of a research assistant. While the three candy rewards remained constant throughout all of the testing, the fourth option varied across participants, due to compatibility issues with the dispensers. Goldfish crackers, peanuts and Cheerios were also used with some participants, and for this reason, the fourth reward is described here as the “other” option. At each offer zone, the movie screens were replaced with a virtual gumball dispenser. Instructions were modified accordingly, describing each zone as a candy shop. Delays were described as the time required to prepare the reward for delivery, rather than the loading time (as in the Movie Row task).

Dispensers were controlled using an Arduino running custom software to interface with the virtual navigation task. Each dispenser was fitted with an infrared detector to verify reward delivery, and rewards were delivered into small plastic magazines, which were also fitted with infrared detectors (to assess reward retrieval by participants).

Participants initially were allowed to sample each of the four rewards (presented outside of the task), and asked to rate their enjoyment of the reward on a scale of 0 to 6 (0 = Not at all, 2 = Slightly, 4 = Quite a bit, 6 = Extremely), and to rank the four rewards from favorite to least favorite. After the task, participants completed a post-task ranking of the rewards and were asked to describe how they selected which food offers to accept and skip, similar to the Movie Row task. Rewards that participants earned, but which were not consumed, were weighed and given to the participant after completion of the task.

#### Delay-Discounting Surveys

##### Monetary Choice Questionnaire

A 21-item version of the Monetary Choice Questionnaire (Kirby et al., 1999) was administered during a set of screening and initial surveys. Reward magnitudes and delays were taken from Wang et al. (2018). For each item, participants chose to either accept a small immediate offer, or to wait the specified time for a larger reward.

##### Food Choice Questionnaire

The 27-item version of the Food Choice Questionnaire (Hendrickson et al., 2015) was administered for a subset of participants before the MCQ. For each item, participants chose between hypothetical food rewards of different magnitudes (specified as a number of bites of one's favorite food). In the original description of the survey, participants were presented with a physical cube to represent the size of one bite (5/8 of an inch, or 1.59 cm). For online administration, the survey was adapted to omit this physical cue, and participants were instructed to “*Please imagine that each bite of food is equal to the size of a cube that is half of an inch tall (about 1.25 centimeters tall)*.”

#### BMI and Smoking Status

Body mass index (BMI) and smoking status were available for a subset of participants (Supplementary Table 2). Self-reported BMI was calculated from self-reported height and weight (or for individuals who were pregnant, from estimates of weight before their pregnancy). Using the Center for Disease Control criteria (www.cdc.gov/obesity/adult/defining.html), analyses using BMI were conducted comparing underweight/healthy individuals (BMI < 25) to overweight/obese individuals (BMI ≥ 25). Participants were classified as smokers if they reported currently smoking cigarettes or using e-cigarettes (vaping), and as non-smokers if they reported using no nicotine products (cigarettes, e-cigarettes, smokeless tobacco, nicotine gum, or nicotine patches).

### Procedure

Participants were tested in-person at Wabash College (for some Movie Row sessions and all Candy row session) or online (some Movie Row sessions). Participants tested on the Movie Row in the laboratory completed the task in a computer lab on PCs divided by partitions. Headphones were provided for Movie Row, so that participants could hear the audio presented with each video.

### Analysis

Analyses were conducted in R. Linear mixed-effects models were fit using the MCMCglmm package (Hadfield, 2010), and categorical variables (gender, smoking status, BMI group, offer value type) were deviation coded (−0.50 vs. +0.50). ANOVAs were conducted using the ez package (Lawrence, 2016).

#### Delay Thresholds

The participants' threshold for deciding to watch or skip videos was estimated by fitting their decisions to stay (= 1) or skip/quit (= 0) as a function of reward type and the delay offered on each trial. Decisions were padded with one stay (1) at 1 s less than the minimum delay offered and one skip (0) at 1 s longer than the maximum delay offered, to account for cases where participants accepted or rejected all of the offers. Fits were performed using a Heaviside function, using a leave-one-out approach in which a threshold for each trial was calculated using every other offer for the same reward type, to calculate the trial-specific threshold (Abram et al., 2019a); thresholds then reflected the point at which the subject had a 50/50 chance of choosing to stay or skip an offer based on the category and delay. Analyses of value used the difference between the offer on the current trial and the trial-specific delay threshold. The average across the trial-specific delay thresholds within a reward type was used as the participant's overall delay threshold for each reward category. To account for cases in which participants preferred long delays, rather than short delays, the Heaviside function was also fit to the inverse of the choice behavior. The error (number of trials that deviated from the predicted outcome) was calculated for both fits. A participant was defined as having a normal delay threshold for a reward category if the average error associated with the Heaviside fit was lower than that of the inverse fit. A participant was defined as having a preference for long delays if the error associated with the Heaviside fit was higher than that of the inverse fit. In the special case where participants skipped/accepted all or nearly all of the offers, the error of both fits would be equal, and these special cases were classified as full-stay (if participants accepted at least 75% of all of the offers for the reward type) or full-skip (if the participant skipped at least 75% of all of the offers for the reward type). For all other cases, the reward type was considered to be unclassified (to lack a delay threshold).

#### Magazine Entries

Breaks of the magazine infrared beam were used to assess retrieval of rewards in the Candy Row task. Breaks lasting between 50 ms and 10 s were scored as a retrieval, and the number of retrievals and duration of the entry were recorded for each trial in each magazine. As participants often did not consume all of the rewards earned, and the infrared sensor could become obstructed by the excess foods, analysis of magazine entries was restricted to the first 20 min of the test session.

#### Offer Zone

After version 1 of the Movie Row task (which lacked a clearly defined offer zone), several measures were calculated for each trial on behavior observed from the time the offer was presented (as the participant entered the offer zone), until the participant crossed out of the offer zone (moving on to the next reward zone, or moving toward the platform). The measures examined were latency to leave the offer zone, distance traveled in the offer zone, total rotation, pausing, rotation reversals, and entry bias (position of the participant when entering the offer zone, relative to the hallway center). Behavior after re-entry to the offer zone on a single trial were not included in these measures. Offer zone behavior on trials with decision latencies longer than 15 s were removed. Behavioral measures (except rotation reversals) were normalized by first using a log_10_ transformation to remove a strong positive skew (toward long times/distances/rotations), then z-scoring (within session) to remove the effects of gender and age (see Supplementary Figure 2B), which may be related to differences in factors such as video game experience.

#### Decision Latency and Distance Traveled

Decision latencies were defined as the time spent in the offer zone (after the participant started moving in Versions 3-4, in milliseconds), and the integrated distance traveled until the participant left the offer zone (in arbitrary units).

#### Total Rotation and Rotation Reversals

The amount of rotation in the offer zone was calculated as the sum of the difference in heading direction for all position samples from when the offer was presented until the participant left the offer zone. The number of reversals of rotation direction in the offer zone was also scored. Rotation measures on trials in which participants rotated more than 360 degrees in the offer zone were removed.

#### Pausing

Pausing in the offer zone was defined as the total amount of time (in milliseconds) participants stopped moving (no position changes or rotation).

#### Entry Bias

After Version 1, entry bias was assessed as the location where participants entered the offer zone, relative to the middle of the pathway (defined as an entry bias of 0). This entry was scaled to the width of the hallway, and values ranged between−50% (toward the participant's left and the reward location, 50% of the hallway width) to +50% (toward the participant's right and the exit used to skip the reward).

#### Reaction Time

In Versions 3-4, the time from when the offer was presented until the participant began moving was defined as the reaction time. Reaction times were log_10_ transformed and z-scored within-session. Data was not analyzed for trials where this measure exceeded 15 s.

#### Regret

Following the procedure of Steiner and Redish (2014), trials were classified as regret-inducing when participants were presented with an offer that was above their delay threshold, after skipping or quitting an offer at the previous zone which was below their delay threshold (thus receiving a low-quality offer after passing up a higher quality offer). These regret trials were compared to two control conditions, Control-1: when participant received an offer that was above their delay threshold, and had previously accepted an offer which was below the delay threshold for the previous zone, and Control-2: when participant received an offer that was above their delay threshold, and had previously skipped or quit an offer which was above the delay threshold for the previous zone.

#### Sunk-Cost Analysis

Following the procedure of Sweis et al. (2018a), the effects of sunk-costs were estimated using relationship between the probability of completing a trial (once the loading bar was started) as a function of the amount of time remaining in the delay. Across participants, the percentage of trials in which participants waited the entire duration of the delay was calculated as a function of the initial delay (for offers between 1 and 29 s). To assess the impact of sunk-costs, the likelihood of completing a given delay was compared to trials in which participants had invested some amount of time (making an investment by waiting a minimum amount of time). For example, to estimate the effects of sunk costs after participants had invested 5 s, all trials in which participants started the delay and waited at least 5 s were selected. Then, the percentage of trials in which participants completed the remainder of the delay was calculated, as a function of the amount of time remaining, over delays from 1 to 23 s (corresponding to initial offers of 6–29 s, see **Figure 6A**).

#### Analyses Involving Gender

Undergraduate participants were recruited from a campus with an all-male student body, so analyses involving gender were conducted with the full dataset, and after excluding the all-male undergraduate samples. Any case where the results obtained with the full dataset were not replicated in analyses excluding the all-male samples are noted in the text.

## Results

### Movie and Candy Row Tasks

Across the participants who completed the Movie and/or Candy Row tasks (and received at least 40 offers in the test phase, *n* = 326), many participants demonstrated the expected pattern of accepting offers with short delays and rejecting offers with long delays, and were more willing to wait for some types of movies or foods (e.g., Figures 1E–G). In some cases, participants accepted the majority or very few of the offers of a given reward type (e.g., full-skip behavior shown in Figure 1E for Accident videos), or showed a preference for long delays. Figure 1H shows the distribution of each response pattern (preferring short delays, preferring long delays, full-stay, full-skip) across participants. Across versions and samples, preferences for long delays were more common when participants were tested online, but the majority of participants demonstrated a preference for short delays for at least one of the four reward types tested.

**Figure 2:**
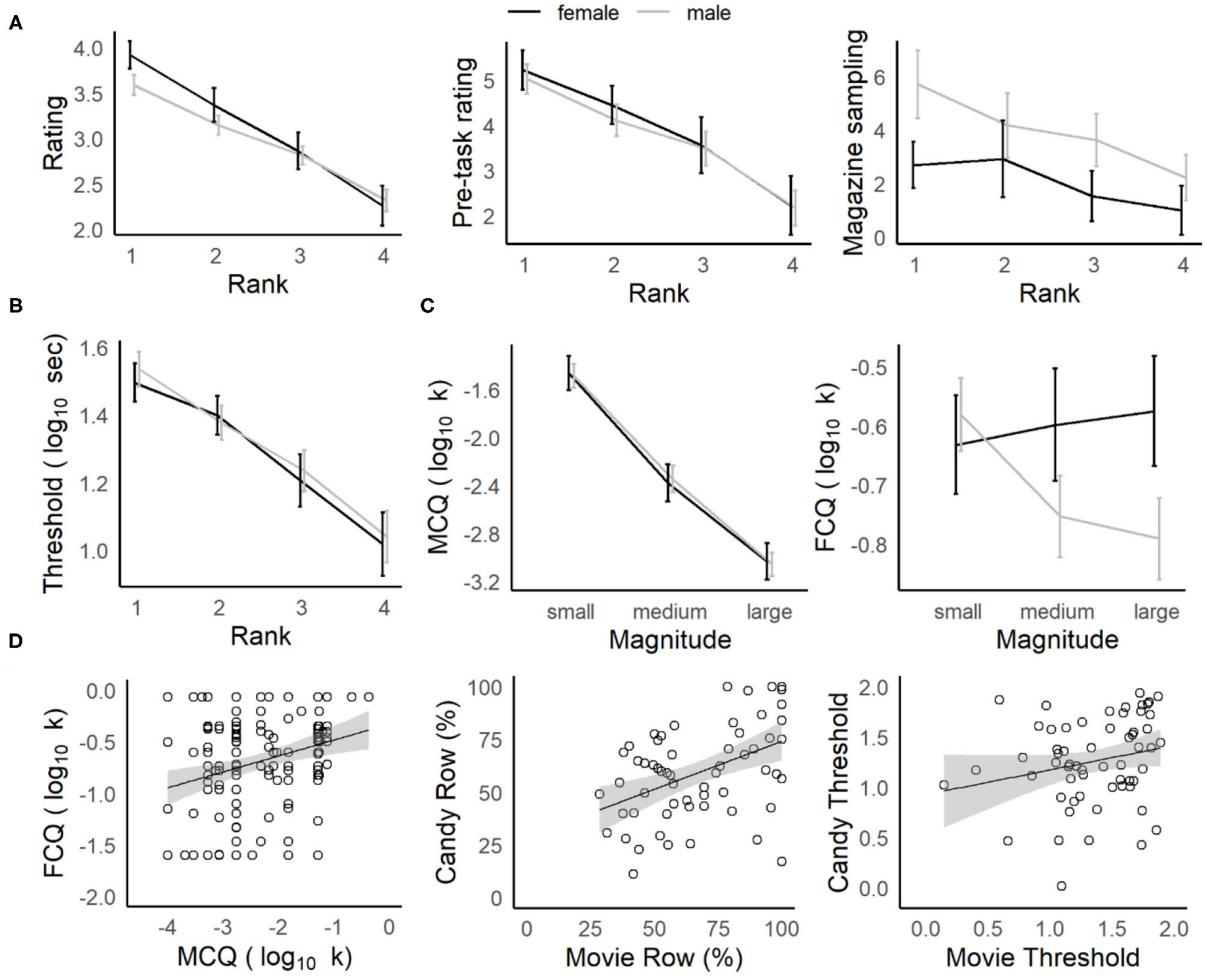
Characterizing navigation tasks and comparison to delay-discounting. **(A)** Stated vs. revealed preferences. Average star ratings (*left*) in the Movie Row, and pre-task enjoyment ratings (*middle*) in the Candy Row decreased as a function of the post-experiment ranking. Participants were more likely to retrieve their favorite food rewards on the Candy Row (*right*). **(B)** Delay thresholds decreased significantly across ranks (favorite to least favorite). **(C)** Delay-discounting (k) for hypothetical money rewards (*left*) did not differ by gender, and decreased as a function of reward magnitude. For hypothetical food rewards (*right*), males showed a similar sensitivity to magnitude, while females did not. **(D)** Comparison of navigation tasks and delay-discounting measures. Overall delay-discounting for hypothetical money and food rewards was correlated (*left*) in the survey measures, as was the number of actual food and movie rewards earned (*middle*) and a similar trend was seen for log-transformed delay thresholds (*right*).

While individuals varied in their preference for specific reward types (on the basis of ratings, post-task rankings, the proportion of offers accepted, delay thresholds, etc.), gender differences were observed across the reward categories (described in the Supplementary Material and Supplementary Figure 1), consistent with reports for the Web-Surf task (Abram et al., 2016). When behavior was analyzed by post-task rankings for each reward type (ranked from most to least favorite), there were strong relationships between rank and other measures of stated preference (post-video ratings on the Movie Row, pre-task enjoyment ratings and magazine sampling on the Candy Row, see Figure 2A) and the participants' revealed preferences (the proportion of rewards earned and delay thresholds, see Figure 2B). In a separate ANOVA for each dependent measure and reward set (original Web-Surf videos, new videos, candy/snacks), with Rank as a within-subjects factor and Gender as a between-subjects factor, there was a significant main effect of Rank for each measure (ps < 0.001, $\eta_{p}^{2}$s > 0.09).

Additionally, females gave higher star ratings than males did to their top ranked video type for the original Web-Surf videos [Rank × Gender interaction: *F*_(3, 891)_ = 7.2, *p* < 0.001, $\eta_{p}^{2}\;$ = 0.024, however the interaction was not significant when the all-male undegraduate sample was omitted, *p* = 0.21, $\eta_{p}^{2}\;$ = 0.001], while males were more likely to sample the magazine (and presumably retrieve rewards) in the Candy Row task [Gender: *F*_(1, 57)_ = 3.2, *p* = 0.023, $\eta_{p}^{2}\;$ = 0.078, however the effect of Gender was not significant when the all-male undegraduate sample was omitted, *F* < 1, n.s.]. Other main effects and interactions were not significant (all Fs < 1.7, ps > 0.22, $\eta_{p}^{2}$ < 0.026).

These results indicate that there was a strong correspondence between the participants' stated reward preferences and their actual choice behavior. And, while females rated videos in their favorite category higher than males for the original Web-Surf videos, and males were more likely to reach into the food magazines in the Candy Row task, these relationships were reduced when the all-male undergraduate sample was omitted, and there were no significant gender differences in revealed preferences (delay thresholds and the proportion of rewards earned).

### Relationship to Delay Discounting

Discounting rates decreased as a function of the reward magnitude for the MCQ and for males in the FCQ (Figure 2C, described in the Supplementary Materials). Overall, discounting rates (log_10_ k) for the MCQ and FCQ were positively correlated in the subset of participants who completed at least one Row task [*r*_(169)_ = 0.26, *p* < 0.001, see Table 1 and Figure 2D, left], indicating that participants who were more willing to wait for hypothetical monetary rewards were also somewhat more willing to wait for hypothetical food rewards. And, the percentage of rewards earned in the Movie Row and Candy Row tasks were positively correlated in the subset of participants who completed both [*r*_(58)_ = 0.45, *p* < 0.001]; and a similar trend was observed for delay thresholds [*r*_(58)_ = 0.22, *p* = 0.089], averaged across the log_10_ transformed delay thresholds calculated within each reward type (Figure 2D, middle and right). However, delay thresholds and the percentage of rewards earned on the Movie and Candy Row tasks were not significantly related to delay-discounting rates derived from the MCQ and FCQ surveys (all *p*s > 0.10). These results indicate that willingness to wait for hypothetical monetary and food rewards in the survey measures was not predictive of actual willingness to wait for video and food rewards in the experiential foraging tasks.

**Table 1:** Correlation between discounting rates (log_10_ k) and row task performance.

**Variables**	**1**.	**2**.	**3**.	**4**.	**5**.	**6**.	**7**.	**8**.	**9**.	**10**.
1. MCQ k	–									
2. FCQ k	0.26^**^	–								
3. MR delay threshold	−0.02	0.12	–							
4. CR delay threshold	−0.08	−0.01	0.22	–						
5. MR % earned	0.02	0.12	0.89^**^	0.30^*^	–					
6. CR % earned	−0.09	0.11	0.38^**^	0.92^**^	0.45^**^	–				
7. Age	−0.14	0.10	0.06	0.03	0.13	0.10	–			
8. Gender	0.02	−0.11	0.05	−0.08	0.04	−0.07	−0.38^**^	–		
9. BMI	0.01	0.04	−0.02	0.10	0.03	0.08	0.22^**^	−0.13^*^		
10. Smoker	0.19^**^	0.10	0.04	0.16	0.06	0.33	−0.04	−0.02	−0.03	
*M*	−2.34	−0.68	1.28	1.27	62.1	60.3	35.1		28.4	
*SD*	0.87	0.51	0.33	0.43	23.6	23.0	13.2		7.6	
*N*	338	171	317	61	324	61	274	360	626	208

*MR, Movie Row; CR, Candy Row; MCQ, Monetary Choice Questionnaire (log_10_ k); FCQ, Food Choice Questionnaire (log_10_ k); % earned = percentage of rewards earned in the Movie/Candy Row tasks. Delay thresholds were log-transformed. Gender was coded as 1 = female, 2 = male. Smoking status (Smoker) was coded as 0 = non-smoker, 1 = smoker. Of the participants, 326 of 807 were female, and 160 of 627 for whom smoking status was assessed were smokers*.

* *p < 0.05*,

** *p < 0.001*.

### Relationship to Smoking and BMI

**Figure 3:**
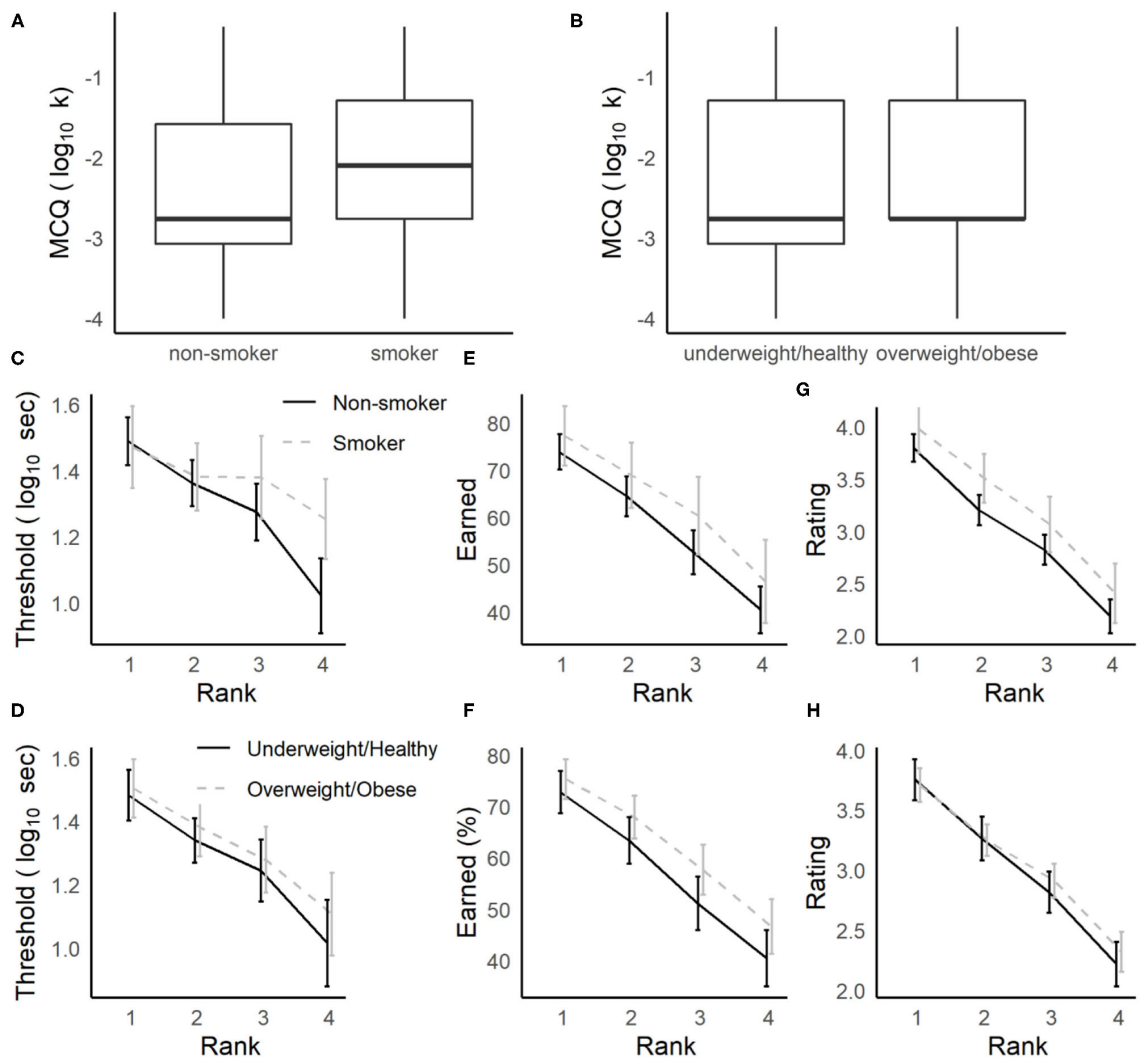
Relationships between delay-discounting and foraging measures to smoking status and obesity. **(A,B)** Smokers more strongly discounted hypothetical money offers in the MCQ **(A)**, and overweight/obese individuals did not **(B)**. **(C–H)** During foraging, delay thresholds were not strongly related to smoking **(C)** or BMI **(D)**. Smokers **(E)** and overweight/obese individuals **(G)** tended to earn more rewards (non-significant), but in smokers this trend could be explained by higher subjective ratings of the rewards on the Movie Row task **(G)**, while BMI group differences were not related to movie ratings **(H)**.

For participants who completed the delay discounting measures as part of the screening surveys (MCQ and FCQ), we expected that smokers and those who report high BMIs (≥25, falling in the overweight or obese range, Jarmolowicz et al., 2014) would have higher discounting rates (k) compared to non-smokers and those who report BMIs <25. In separate ANOVAs, overall discounting rates (log_10_ k) for the MCQ and FCQ were analyzed for participants who completed at least one Row task session, with Gender, Smoker (non-smoker, smoker) and BMI (<25: underweight/healthy, ≥25: overweight/obese) as between-subjects factors. For the MCQ, smokers had significantly higher discounting rates compared to non-smokers [*F*_(1, 169)_ = 4.2, *p* = 0.042, $\eta_{p}^{2}\;$ = 0.024, see Figure 3A], while the differences between overweight/obese and underweight/healthy individuals in this sample were not significant (see Figure 3B, all other main effects and interactions, ps > 0.084, $\eta_{p}^{2}$s < 0.014). For the FCQ, no main effects or interactions with smoking status or BMI group were significant (*p* > 0.072, $\eta_{p}^{2}\;<$ 0.027).

While the overall discounting rates for the MCQ were elevated for smokers, there were no strong differences observed in delay thresholds or proportion of rewards earned on the Movie and Candy Row tasks among participants for whom smoking status and BMI were available (Supplementary Table 2). And, any trends observed in these samples were in the opposite direction, where smokers were more willing to wait for rewards on the Movie Row task (see Figures 3C–F). In separate three-way ANOVAs, we analyzed log_10_ transformed delay thresholds and the percentage of rewards earned as a function of Rank as a within-subjects factor, Gender as a between subjects-factor and either Smoker or BMI group as a second between-subjects factor. Analyses were restricted to the Movie Row task, due to the small number of Candy Row sessions available. These models revealed no main effect or interactions with Gender (ps > 0.45, $\eta_{p}^{2}\;<$ 0.003), nor any main effects or interactions of BMI group (ps > 0.27, $\eta_{p}^{2}$ < 0.017). Though the main effects for smoking status were not significant, smokers tended to have somewhat higher delay thresholds [*F*_(1, 138)_ = 3.2, *p* = 0.076, $\eta_{p}^{2}$ = 0.023] and to earn more rewards [*F*_(1, 175)_ = 3.7, *p* = 0.057, $\eta_{p}^{2}\;=$ 0.021].

These results indicate that, in contrast to the results for the MCQ, neither smoking status nor BMI group were significantly related to willingness to wait for rewards. And, any trends in these data suggested the smokers were willing to wait longer for actual video or food rewards in a foraging task. This trend, even if validated in another sample, may be driven by differences between smokers and non-smokers in their subjective enjoyment of the video rewards. Analyzing the post-video ratings made on the Movie Row task using an ANOVA with Smoker and Gender as between-subjects factor, and Rank as a within-subjects factor, smokers gave significantly higher ratings to the videos they watched than did non-smokers [Figure 3G, *F*_(1, 172)_ = 3.9, *p* = 0.049, $\eta_{p}^{2}$ = 0.022]. There was also a main effect of Rank (*p* < 0.001), but in the subset of participants for whom a smoking status was available, there were no main effects or interactions with Gender (ps > 0.14, $\eta_{p}^{2}$ < 0.012), and no significant relationships to BMI group were obtained (Figure 3H, ps > 0.23, $\eta_{p}^{2}$ < 0.007). Thus, it is possible that if smokers are somewhat more likely to accept offers on the Movie Row task, this could be driven by differences in enjoyment of rewards in the task.

### Deliberation

Rats and mice demonstrate vicarious-trial-and-error (VTE) in the offer zone, when deliberating over which offers to accept (Steiner and Redish, 2014; Sweis et al., 2018c). Steiner and Redish first demonstrated that VTE (measured as the integrated absolute angular change in the orientation of motion of the head) was sensitive to value (the difference between the delay threshold and the delay offered on a given trial) in rats tested on Restaurant Row. For difficult offers (value = 0, for delays close to the threshold), rats and mice spend more time in the offer zone and engage in more VTE, consistent with the proposal that VTE is a behavioral correlate of deliberation that is enhanced for difficult offers. Similarly, humans tested on the Web-Surf task take more time to make a stay/skip decision for difficult offers, where the value = 0 (Abram et al., 2019a). To examine the relationship between deliberation and behavior in humans further, the relationship between value (delay offered on a trial minus the participant's delay threshold) and each behavioral measure was examined. We predicted that more difficult offers (value = 0), would require more deliberative processing, and that these behavioral measures (related to the time taken to make a decision, or the movements while in the offer zone) would be sensitive to value, and peak at value = 0.

**Figure 4:**
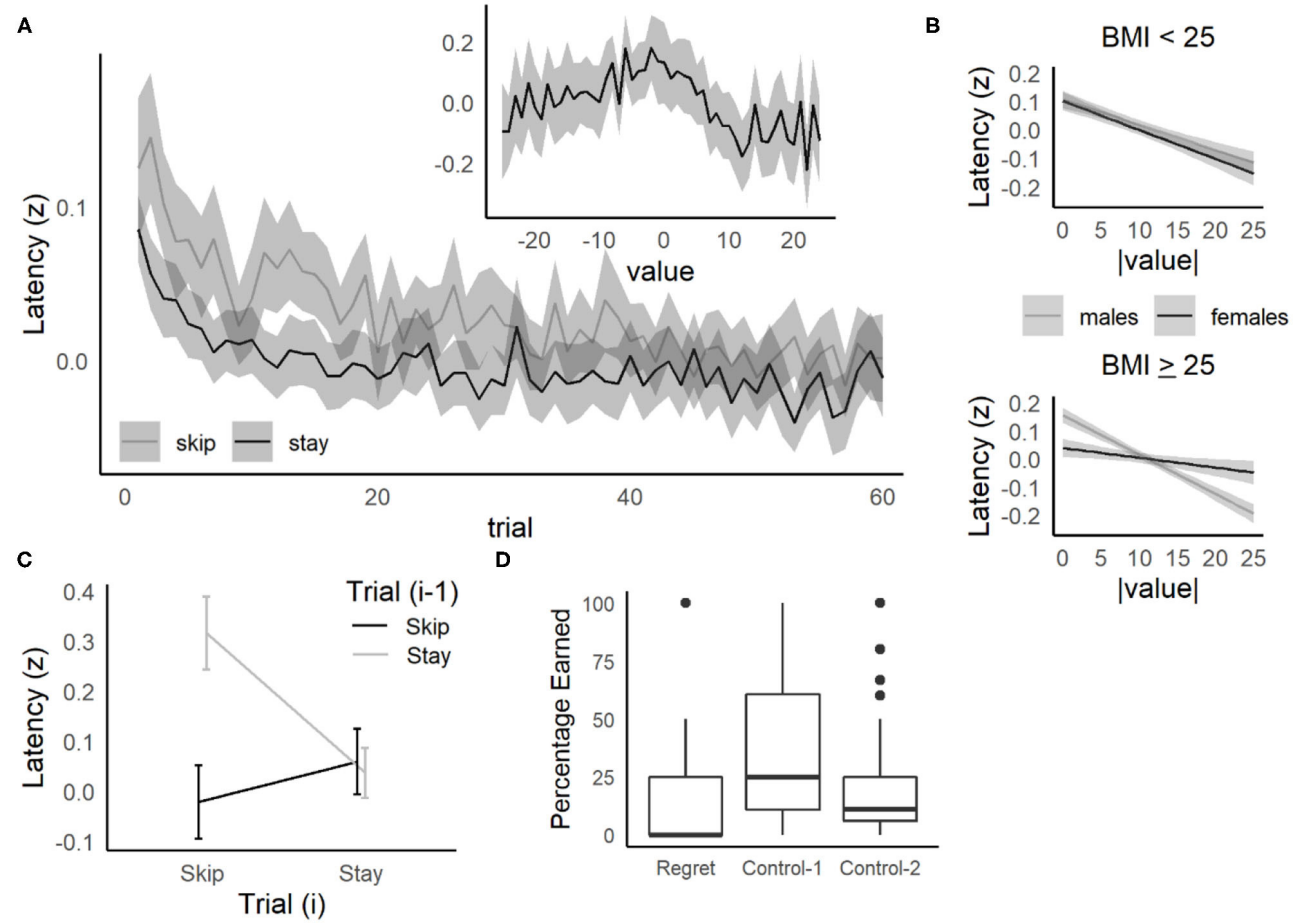
Replication of behavioral results from the Web-Surf and Restaurant Row and relationship to BMI. **(A)** Decision latency relative to trial number and value (threshold—delay). The time taken to make a choice (log-transformed and z-scored) was higher overall for skip decisions, but decreased across trials for both skip and stay decisions. *Inset:* Decision latency was also elevated for difficult decisions (value = 0, offers near threshold). **(B)** Higher BMI females (> 25–overweight/obese) were less sensitive to value compared to males with BMI > 25, and to males and females with BMI < 25. Lines and shaded area represent estimated means and 95% confidence intervals based on the regression model. **(C)** Sequential choice behavior. Participants were slower to accept an offer (on trial *i*) after previously skipping an offer (on trial *i-1*), compared to other sequences (Skip/Skip, Stay/Stay, or Stay/Skip). **(A,C)** For each plot, data were averaged within participant, then means and confidence intervals were calculated across participants. Lines in *C* indicate means, and errorbars/shaded areas **(A,C)** indicate 95% confidence intervals. **(D)** Regret. Participants were less likely to accept low-value offers (> threshold) in regret-inducing conditions (previously rejecting a high-value offer) compared to control conditions (*control-1*: previously accepted a high-value offer, *control-2*: previously skipped a low-value offer).

For decision latency, the pattern expected for deliberation was observed (Figure 4A, inset), with the highest latencies observed for the most difficult offers (peaking as the delay offered approached the participant's threshold for that reward type). A linear mixed-effects model was fit with decision latency (*z*-scored) as the dependent variable, absolute value of the offer (|value|: 0–25 s) as a continuous predictor, and value type (deviation coded as −0.50 = < threshold vs. +0.50 = > threshold) and their interactions included as fixed-effect independent variables and participant as a random effect [*z*_*latency*_ ~ *|value|* + *value type* + *gender* + *|value|:value type* + *|value|:gender* + *value type:gender* + *(1|participant)*]. Decision latencies increased significantly for difficult offers, as value approached 0 (Supplementary Table 3, *p* < 0.001, p-adj = 0.004), and this relationship was stronger for males compared to females (*|value|:gender, p* = 0.014, *p*-adj = 0.025).

There was also a significant *value type*: *gender* interaction, where males had higher decision latencies for poor offers (< threshold) compared to good offers (> threshold), while females showed the opposite pattern. This pattern was not simply related to gender differences in entry bias (the position the participant entered the offer zone relative to the center of the hallway), as this did not differ on average between males and females [*t*_(285)_ = 0.8, *p* = 0.44, *d* = 0.10], and including a rescored measure of entry bias (the position that the participant entered the offer zone relative to their eventual choice, which affected the time required to exit the offer zone) did not eliminate this interaction.

Both the *value type*:*gender* and *|value|:gender* interactions remained significant when the all-male undergraduate sample was excluded from the regression. The *|value|:gender* interaction was reduced when the sample was restricted to those ages 40 and younger (with [*n* = 52 females, 112 males] or without [*n* = 52 females, 49 males] the all-male undergraduate sample, ps > 0.48, *p*-adjs > 0.59), but was significant when restricted to those over age 40 (*n* = 45 females, 27 males, *p* < 0.001, *p*-adj = 0.0028). As we did not assess video game experience in this study, one possibility is that these relationships to gender and age may be explained by a confound with previous experience with first-person games, similar to the present task.

**Figure 5:**
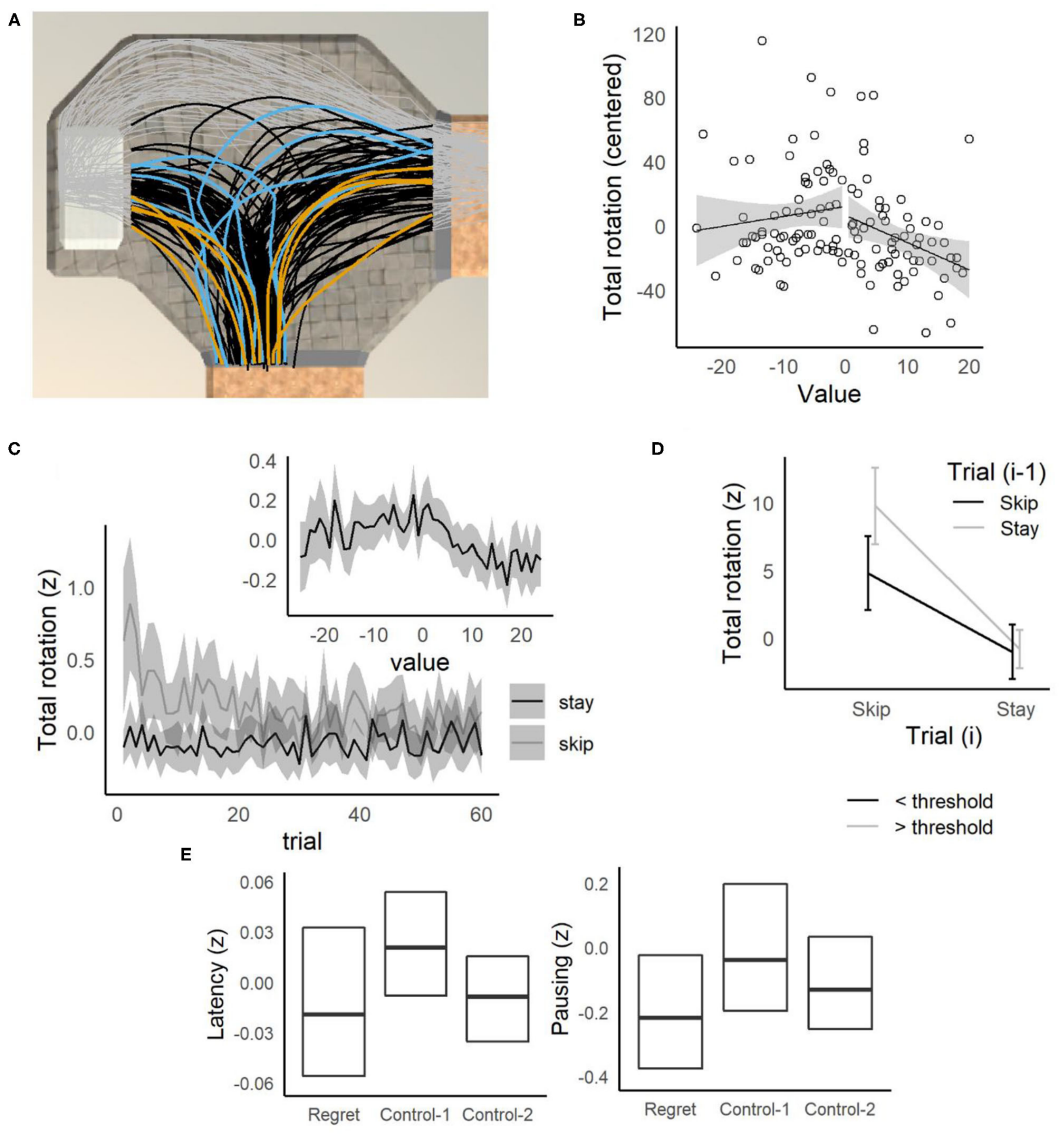
Offer zone behavior. **(A)** Example of position data from one participant (MRM336), rotated to overlay a single offer zone. All position data are shown as gray points, black lines indicate paths taken up to the point where the participant left the offer zone. Randomly selected trials when the participant's choice time was slow (blue) vs. fast (orange) show that the participant tended to take longer paths and make corrections on slow trials. **(B)** Relative to offer value (threshold—offer), total angular rotation was elevated for difficult offers (value = 0) for MRM336. **(C)** Across participants, total rotation was elevated for difficult decisions (value = 0). Total rotation decreased across trials for skip decisions, but not stay decisions. **(D)** Total rotation was higher overall for skip trials compared to stay trials, but highest when participants skipped the current offer after accepting the previous offer. **(E)** Participants made faster decisions on regret trials, and were less likely to pause, compared to control-1 trials.

Longer decision latencies for difficult decisions were also associated with increases in VTE-like behavior (see example in Figure 5B). In separate regressions for other behavioral measures (Supplementary Table 3), a significant relationship to |value| was observed for each measure, indicating that for difficult offers, participants had slower reaction times, and longer decision latencies, where they were not only slower to make decisions but they were also more likely to pause, travel farther, rotate more, and change their direction of rotation (Figure 5C, Supplementary Figure 3). When gender was added to the regressions for these behavioral measures, the patterns observed were similar to those obtained for decision latencies (for the *value type*:*gender* and *|value|:gender* interactions), and the relationship to |value| remained significant.

The tendency for decision latencies to be elevated for difficult offers was also related to BMI, but not to smoking status (Supplementary Table 4). When smoking status (non-smoker vs. cigarette/e-cigarette user) was added to the model, including the 2-way interactions with entry bias and value type, and also the 3-way interactions with gender, there were no significant effects related to smoking status (ps > 0.23, *p*-adjs > 0.44, Supplementary Table 4 provides the coefficients for a model that excludes gender, for which the model estimates were essentially identical).

Adding BMI group (< vs. ≥ 25) to the decision latency regression model did reveal that the |*value*|:*gender*:*BMI group* interaction approached significance (*β* = 0.012, 95% CI = 0.0022, 0.022, *p* = 0.022, *p*-adj = 0.072, Figure 4B). A similar pattern was seen when the all-male undergraduate samples were excluded (*p* = 0.014, *p*-adj = 0.048), as well as a significant *value type:gender:bmi group* interaction (*β* = −0.17, 95% CI = −0.32, −0.017, *p* = 0.030, *p*-adj = 0.049). Separate regressions by BMI group found that both groups demonstrated a significant relationship to |value| (ps < 0.001, *p*-adjs < 0.005, Supplementary Table 4). As shown in Figure 4B, for underweight/healthy individuals, the slope for |value| was similar for males and females. However, for overweight/obese individuals, females specifically showed reduced sensitivity to |value|, suggesting that females with BMI ≥ 25 may show less deliberation when presented with difficult offers (close to threshold).

### Sequential Choices

Overall, decision latencies were slower and more VTE was observed when participants made choices that were not consistent with their thresholds (accepting offers above their delay threshold, or rejecting offers below their delay threshold, Supplementary Material and Supplementary Figure 5), suggesting that deliberation is enhanced when participants overrode their preferences. Similarly, Abram et al. (2019a) found that humans tested on the Web-Surf task were slowest to make a decision when skipping a trial after accepting the previous offer, suggesting that deliberation may be enhanced when overriding the default strategy to stay engage with an offer. Similar results were obtained in the Movie/Candy Row tasks, with decision latencies significantly higher on trials where participants skipped an offer after accepting the previous offer (Figure 4C). In a three-factor ANOVA with decision on the current trial *i* (Decision_(i)_: skip, stay) and decision on previous trial *i-1* (Decision_(i−1)_: skip, stay) as within-subjects factors, and Gender as a between-subjects factor, there was a significant main effect of Decision_(i−1)_ [*F*_(1, 242)_ = 46.1, *p* < 0.001, $\eta_{p}^{2}$ = 0.024], but not Decision_(i)_ [*F*_(1, 242)_ = 1.9, *p* = 0.17, $\eta_{p}^{2}$ = 0.005]. Importantly, there was a significant Decision_(i)_ × Decision_(i−1)_ interaction [*F*_(1, 242)_ = 29.1, *p* < 0.001, $\eta_{p}^{2}$ = 0.015]. Decision latencies were slower when participants skipped an offer after accepting the previous offer (Stay/Skip) compared to Skip/Skip [*t*_(187)_ = 7.5, *p* < 0.001], Stay/Stay [*t*_(187)_ = 4.3, *p* < 0.001] and Skip/Stay [*t*_(187)_ = 4.5, *p* < 0.001] conditions.

However, these results were qualified by a significant Gender × Decision_(i)_ × Decision_(i−1)_ interaction [*F*_(1, 242)_ = 4.2, *p* = 0.042, $\eta_{p}^{2}\;$ = 0.002], but which was not significant when the all-male undergraduate sample was removed [*p* = 0.62, $\eta_{p}^{2}$ < 0.001]. The Decision_(i)_ × Decision_(i−1)_ interactions were significant in both this sample and the all-male sample, tested with a separate ANOVA excluding gender (ps < 0.002, $\eta_{p}^{2}$s > 0.008), indicating that this result was not limited to one of the samples, and did not depend strongly on gender. On Stay/Skip trials, participants also traveled farther on average, paused longer, and were more likely to reverse their direction of rotation (described in Supplementary Material, see Figure 5D and Supplementary Figure 6) compared to Skip/Skip trials, indicating greater levels of VTE on Stay/Skip trials.

### Sunk-Costs

To determine if participants demonstrated a sunk-cost effect on the Movie and Candy Row tasks [as in Sweis et al. (2018a)], we examined how the probability of completing the entire delay for an offer was related to the amount of time already invested. After starting the delay, participants on the Movie Row and Candy Row tasks were very unlikely to quit compared to published data from the Restaurant Row (but similar to published data from the Web-Surf task). Across the samples tested after version 1 of the Movie Row task (which lacked a well-defined waiting zone), participants quit before the delay was completed on 0.7% of all trials in which participants stepped onto the platform (0.4% of all trials), which is lower than rates for both the Web-Surf task and Restaurant Row. After reaching the platform, participants were highly likely to initiate the loading bar (in versions 2 and 3 of the Movie Row, which had a stricter requirement for starting the loading bar, participants started the loading bar on 99.6% of trials in which they stepped to the platform). Also, only 62 of the 296 participants (21%) tested after version 1 ever quit a trial after stepping onto the platform during their first Movie or Candy row task session. Among participants who quit on at least one trial after initiating the delay, participants quit on average 3.3% (SD = 2.3%, range = 1.5–14.3%) of trials in which they started the loading bar. The low quit rate on the Movie Row and Candy Row tasks may be related to the high response requirement to start the delay in versions 2-3 (participants must move to and stand on a small platform and look directly at the screen). However, quit rates remained low (M = 0.5%, SD = 1.1%) in version 4 and in the Candy Row, where the platform area was expanded and the requirement to initiate the delay was relaxed. It is also possible that low quit rates indicate a lack of awareness on the part of the participants that quitting during a delay was allowed (in spite of the instructions provided).

**Figure 6:**
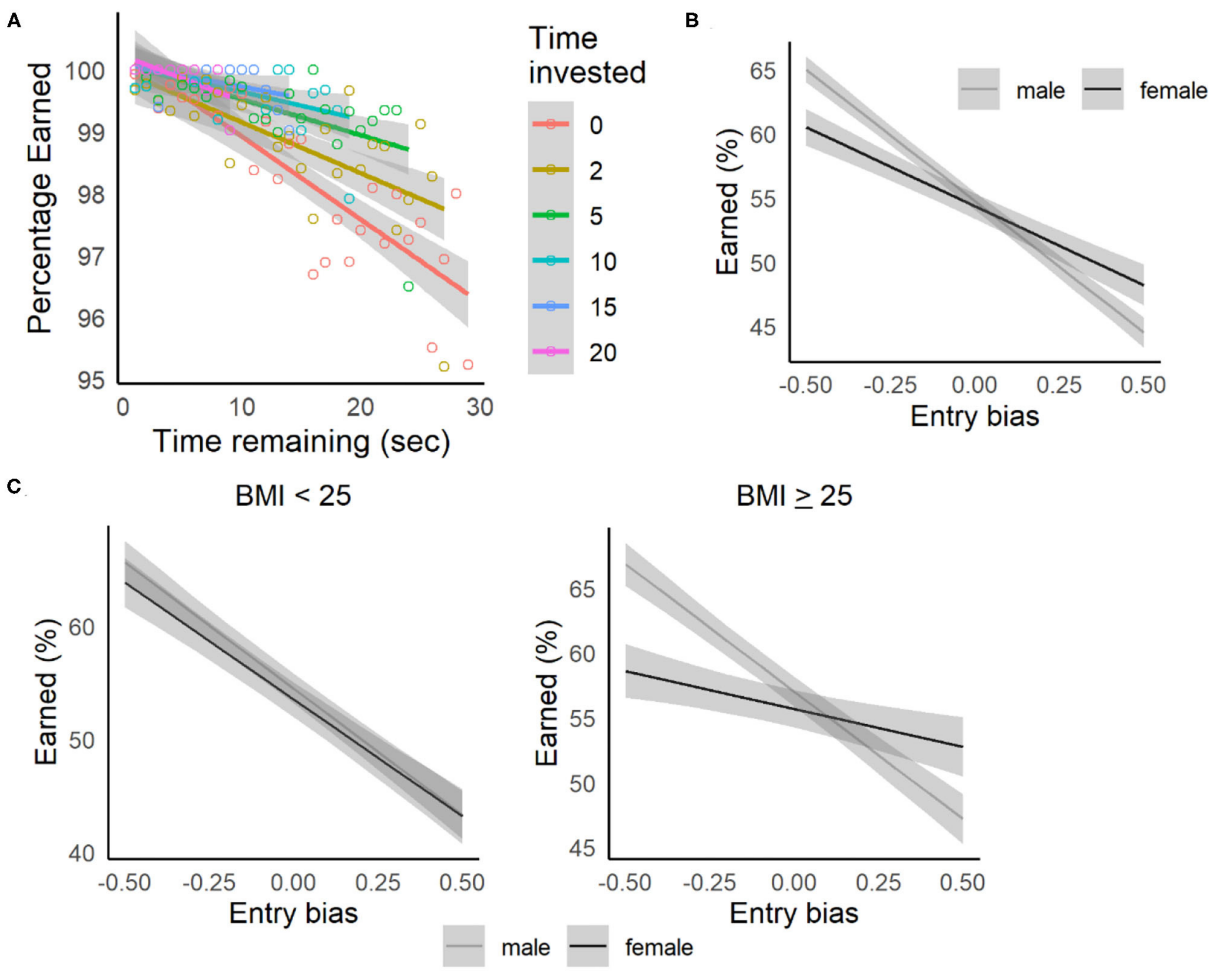
Sunk-cost relationships. **(A)** Replication of sunk-cost results from the Restaurant Row and Web-Surf tasks. Quit rates (leaving before the delay was completed) increased as a function of delay (time invested = 0 seconds). The probability of staying for the remainder of the delay increased as a function of amount of time invested (investments of 2–20 s shown). Shaded areas indicate the 95% confidence intervals, color-coded by the amount of time invested. **(B)** Percentage of rewards earned by gender, estimated as a function of entry bias (relative to the center of the pathway). Participants were more likely to accept rewards when entering to the left of center, and more likely to skip them when entering to the right of center, with females showing a weaker sensitivity to this bias, especially for those with BMI ≥ 25 **(C)**. Lines and shaded area represent estimated means and 95% confidence intervals based on the regression model.

While quit rates were low on the Movie and Candy Row tasks, we did observe evidence that the decision to quit was sensitive to sunk-costs. As shown in **Figure 6A**, the likelihood of completing the delay decreased as a function of the initial delay (assessed across all participants, 95% CI for the slope = −0.00078, −0.00025). However, as participants have invested time (waiting during the delay), this relationship to delay weakens as a function of the amount of time invested (after completing 5 s, 95% CI = −0.00048, 0.00003, after 10 s, CI = −0.00023, 0.00013). While quit rates were low, once participants had invested several seconds waiting during a delay, they were more likely to complete the remainder of the delay, consistent with previous reports from the Web-Surf task and Restaurant Row.

The low quit rates observed across the four versions of these tasks may indicate that by the point at which participants reached the platform and initiated the delay, they had already made a substantial investment, and that their behavior was already influenced by sunk-costs (involved in making a choice). In that case, a better measure of sensitivity to sunk-costs might be observed earlier in the process of deciding to accept or reject an offer. On the Movie and Candy Row tasks, another sunk-cost (besides the amount of time invested in waiting during the delay) can be investigated at the point at which participants receive an offer after entering the offer zone. As participants approached an offer zone, no constraints were placed on the position of the participants within the width of the hallway leading into the offer zone. On many occasions, participants entered with a bias to the left (toward the waiting zone) or right (toward the exit) of the center of the hallway (for example, see Figure 5A where the participant shows an overall bias toward the waiting zone). Based on the average movement speed, ~1.25 s were required to cross the full width of the hallway. As the median decision latency (calculated within, then across participants) to make a choice when participants entered near the center of the hallway was 2.7 s, a bias in the location where participants entered the offer zone (toward the left or right of center) would substantially impact the amount of time required to make a choice. The location that a participant enters the offer zone can thus be considered a sunk-cost in that participants made their entry before knowing what the quality of an offer they would receive, and the cost of their subsequent decision was strongly impacted by their entry bias.

To assess the impact of entry bias on stay/skip decisions, a linear mixed-effects model was fit with Choice (1= stay, 0 = skip) as the dependent variable, value (deviation coded as −0.50 = < threshold, +0.50 = > threshold) and entry bias (−50% to +50% of the hallway width), gender, and the interactions of these factors as fixed-effect independent variables and participant as a random effect [*Choice* ~ *value type* + *entry bias* + *gender* + *value type:entry bias* + *value type:gender* + *entry bias:gender* + *(1|participant)*]. As expected, participants were more likely to accept high value offers (delay < threshold) compared to low value offers (delay > threshold, see Supplementary Table 5, *p* < 0.001, *p*-adj = 0.002). The probability of accepting an offer also declined as the entry bias increased, and participants entered the offer zone to their right (*p* < 0.001, *p*-adj = 0.002). There was also a significant *entry bias:gender* interaction, where males showed a stronger sensitivity to entry bias overall (Figure 6B). This gender difference may represent a confound with factors such as video game experience, which was not assessed in the present study. Across gender, when comparing estimates of the percentage of rewards earned based on this model (using percentage of rewards earned as the dependent) for an entry bias near 0 (entering at the center of the hallway) to when participants were biased toward the left by 25% (halfway between the center of the path and the waiting zone), the percentage of poor offers accepted increased 5.2%. And, when participants were biased toward the right by 25% (toward the exit), the percentage of good offers skipped was 3.7% higher. These data support the interpretation that entry bias represents a sunk-cost, and that participants were more likely to reject good offers and accept bad offers if they had invested effort in moving toward the disadvantageous choice (toward skipping on trials with good offers, and toward staying on trials with bad offers) before the value of the offer was revealed.

The relationship of choices to entry bias was also sensitive to BMI and smoking status in these samples. When BMI group (< or ≥ 25) was added to the model, including the 2-way interactions with entry bias and value type, and also the 3-way interactions with gender, the *entry bias:gender:BMI group* approached significance (*β* = 0.12, 95% CI = 0.0037, 0.24, *p* = 0.036, *p*-adj = 0.067). A similar pattern was seen when the analysis removed the all-male undergraduate samples, and was restricted to non-smokers (*p* = 0.030, *p*-adj = 0.058), in addition to observing an interaction between *value type:gender:bmi group* (*β* = 0.091, 95% CI = 0.031, 0.15, *p* = 0.004, *p*-adj = 0.009). Separate models were then fit for each BMI group (*Choice* ~ *value type* + *entry bias* + *gender* + *value type:entry bias* + *value type:gender* + *entry bias:gender* + *(1|participant)*, see Supplementary Table 5). For both males and females, there was a significant *value type:bmi group* interaction (ps < 0.001, *p*-adjs = 0.002), where individuals with self-reported BMI ≥ 25 were more likely to accept poor offers and reject good offers. Females with BMI ≥ 25 showed a weaker sensitivity to entry bias (*entry bias:bmi group* interaction) compared to females with BMI < 25, suggesting that overweight/obese females showed a weaker sunk-cost effect (Figure 6C).

When smoking status (non-smoker vs. smoker/e-cigarette users) was added to the original model instead of BMI group, including the 2-way interactions with entry bias and value type, and also the 3-way interactions with gender, the *entry bias:gender:smoker* was significant (*β* = −0.23, 95% CI = −0.41, −0.054, *p* = 0.014, *p*-adj = 0.046). In separate models fit for males and females (*Choice* ~ *value type* + *entry bias* + *gender* + *value type:entry bias* + *value type:gender* + *entry bias:gender* + *(1|participant)*, see Supplementary Table 5), males demonstrated a significant *value type:smoker* interaction (*p* = 0.008, *p*-adj = 0.014), where male smokers were less sensitive to entry bias than non-smokers. This relationship was related to BMI as well: the interaction was significant for males with BMI ≥ 25 (*β* = 0.26, 95% CI = 0.074, 0.44, *p* = 0.004, *p*-adj = 0.009), but not for those with BMI < 25 (*β* = 0.027, 95% CI = −0.27, 0.33, *p* = 0.89, *p*-adj = 0.89). Together, these results indicate that overall, higher BMI (>25) was associated with reduced sensitivity to entry bias, though this relationship for males was only observed for those who used cigarettes or e-cigarettes.

### Regret

Steiner and Redish (2014) reported several behavioral correlates of regret in rats tested on Restaurant Row, where in regret-inducing situations (receiving a poor offer after skipping a good offer), rats were more likely to accept a poor offer, spent less time consuming the reward, and were likely look back toward the previous (skipped) food location compared to the two control situations. On the Movie and Candy Row tasks, participants were less (rather than more) likely accept a poor offer when compared to the control conditions (Wilcoxon's P, Control-1: *p* < 0.001, Control-2: *p* < 0.001, see Figure 4D). These data suggest that humans, like rats, behaviorally differentiate between regret-inducing and control trials, although there appear to be species or task-related differences (e.g., rats being more likely to accept offers on regret-inducing trials, and humans being less likely to do so).

Additionally, participants were less likely to pause on regret trials (Control-1: *p* < 0.001, Control-2: *p* = 0.002), and compared to Control-1 trials, participants left the offer zone more quickly (Figure 5E *left*, Control-1: *p* < 0.001, Control-2: *p* = 0.34), rotated less (Figure 5E *right*, Control-1: *p* < 0.001, Control-2: *p* = 0.59), less often reversed rotation direction (Control-1: *p* < 0.001, Control-2: *p* = 0.32), and traveled a shorter distance while in the offer zone, (Control-1: *p* < 0.001, Control-2: *p* = 0.32). No significant differences in reaction time were observed between conditions (Control-1: *p* = 0.37, Control-2: *p* = 0.71).

## Discussion

Behavior on the Movie Row and Candy Row tasks largely replicated published behavioral results from rodents (in the Restaurant Row navigation task) and humans (in the Web-Surf experiential foraging task). The current findings demonstrate the utility of these tasks for capturing multiple dimensions of decision-making across species, including deliberation, regret and sunk-costs. Our results with the Movie Row and Candy Row tasks extend the comparison of human and rodent decision-making, by demonstrating that when faced with difficult offers (near threshold) and when acting against one's preferences (skipping high-value offers and accepting low-value offers), humans not only take longer to make a decision, but tend to pause longer, rotate farther, change rotation direction, and travel further. These results demonstrate the existence of vicarious trial-and-error (VTE) behaviors in humans, which share many similarities to rodent behavior during Restaurant Row. Taken together, these findings suggest that VTE during navigation may be a behavioral correlate of deliberation shared across humans and rodents.

Rats and mice take more time deciding to accept or skip difficult offers (close to threshold) during Restaurant Row (Steiner and Redish, 2014; Sweis et al., 2018b). These observations are consistent with the proposal that deliberative processing is computationally slow (time-intensive), involving a search through potential future states to find the best decisions or actions to take (Redish, 2013). In rodents, difficult decisions on the Restaurant Row task are also associated with an increase in VTE (i.e., more pause-and-look behavior), which has been proposed to be a behavioral correlate of deliberation in rodents (Steiner and Redish, 2014). During VTE in rats, neural activity in the hippocampus represents “sweeps” through potential future trajectories (Johnson and Redish, 2007), while activity in the orbitofrontal cortex and ventral striatum represent potential goal locations (Stott and Redish, 2014).

While decision times in humans are also slower when faced with difficult decisions on the Web-Surf task (Abram et al., 2019a), behavioral correlates of deliberation in humans (analogous to VTE) have not been well-described. In visuospatial tasks, primates (human and non-human) show evidence for a visual search pattern (“saccade-fixate-saccade”) that is similar to VTE, in which subject' fixations alternate between targets during difficult decisions [reviewed in Redish (2016)]. Revisitation, a return of fixation to the previous stimulus during the study phase is associated with better subsequent memory for items; further, revisitation is reduced in amnesiacs with hippocampal damage, similar to results in rodent VTE (Voss et al., 2011). During difficult perceptual discriminations, revisitation is also associated with improved performance and increased hippocampal activity (Voss and Cohen, 2017).

While patterns of eye movements, such as the saccade-fixate-saccade pattern, share a number of properties with rodent VTE in rats, it is unclear if humans and other primates demonstrate similar VTE behaviors during navigation that are directly comparable. In rats, VTE was initially characterized in tasks where animals were presented with discrete choices (such as two alleys in a maze, and trained to make difficult discriminations, Muenzinger and Gentry, 1931). At these choices, VTE was characterized generally as a “hesitating, looking-back-and-forth, sort of behavior which rats can often be observed to indulge in at a choice-point before actually going one way or the other” (Tolman, 1948, p. 196–197). Muenzinger (1938) identified two primary patterns behavior that characterized VTE in rats trained in discrimination tasks on a T-maze: “Our criterion for recording *VTE* behavior in any one trial was a facing into one alley before the other one, whether right or wrong, was entered. This alternation in facing the two choice alleys is accomplished in various ways by white rats. The most common way is for the rat to stop at a mid-point between the alleys and turn his head first toward one and then toward the other alley. But he may also approach the entrance to the alley and orient his whole body toward it and then turn and approach the other alley in a similar way” (p. 77). These two patterns of behavior are captured in studies by Redish and colleagues by the metric IdPhi (the total angular rotation as animals pass through an offer zone, or in a fixed window of time after entering the offer zone, Papale et al., 2012; Steiner and Redish, 2012). Although, it is likely that when body position coordinates are used rather than head position coordinates, IdPhi is less sensitive to the first type of VTE behavior described by Muenzinger (when animals look back and forth while paused).

In virtual navigation research with humans, one recent study by Santos-Pata and Verschure (2018) found elevated VTE in a virtual navigation task (applying the IdPhi measure to the rotation of the head of the participant's avatar, and quantifying oscillations in head orientation) early in training and at early, high-cost choice points in a multiple T-maze task. In that task, rotation was controlled using a mouse, while movements were controlled using a keyboard (a common key binding for first-person perspective games presented on laptops or desktop computers). The patterns described by Santos-Pata and Verschure are consistent with the proposal that these behaviors represent VTE during the use of hippocampally-dependent place strategies in navigation. Our results extend this work by examining a range of VTE behaviors in situations which promote deliberation, and demonstrating that behaviors which are comparable to rodent VTE are enhanced when humans appear to be deliberating between options or when acting against their preferences. Our results seem to be most consistent with the second VTE pattern described by Muenzinger (1938), in that participants who took longer to make a choice were likely to make an initial commitment to one option (to stay or skip), then changing direction one or more times before making a final choice (see examples in Figure 5A). In our study, movement was also controlled by using the keyboard to rotate, which may impact the type of VTE that is observed in our task, compared to that of Santos-Pata and Verschure (2018). However, using the keyboard, rather than using a mouse to control orientation, may be preferable for testing participants with a wide range of computer experience, and our results suggest that even with this simpler set of movement controls, we see robust evidence for VTE in the Movie and Candy Row tasks.

Beyond deliberation, the results from the Movie and Candy Row tasks also replicate and extend previous findings from Restaurant Row and Web-Surf task. On the Movie and Candy Row tasks, participants demonstrated consistent preferences for video types, which agreed well with their stated preferences. In participants tested on both tasks (one using videos as rewards, and the other using actual food rewards), performance was correlated, where individuals who were more willing to wait for video rewards tended to also be willing to wait for palatable foods (candy and snacks). While there may be situations in which food is a preferred reward, these results support the generalizability of research using videos as rewards (in the Web-Surf task and Movie Row), an approach that is more suitable for online data collection and testing larger samples.

One species difference noted here was during a regret-inducing situation (receiving a low-value offer after skipping a high-value offer), humans were less likely to accept the low-value offer, and appeared less likely to deliberate (spent less time in the offer zone, rotated less, were less likely to pause and tended to travel a shorter distance, compared to control trials). Compared to control conditions, rats in regret-inducing situations are more, rather than less, likely to accept low-value offers (Steiner and Redish, 2014), and are more likely to look back at the previous reward location and consume the rewards earned more quickly. During regret-inducing trials, activity of neural ensembles in the orbitofrontal cortex and ventral striatum also tended to represent the previous location (where rats had skipped a high-value offer), activity which may be important in reevaluating past decisions to guide future behavior. Interesting, neural representations in humans (assessed by fMRI) are enhanced for the current location (rather than the previous one) on regret-inducing trials (Abram et al., 2019a), the results presented here further support species differences in behavioral and neural processes associated with regret.

Replicating previous research, participants also demonstrated a sunk-cost bias, with participants' likelihood of completing a delay increasing as a function of the amount of time already invested in waiting. A separate measure of sunk-costs was also identified, based on the initial position at which participants entered the offer zone (toward the reward waiting zone, or toward the offer zone exit). While participants on the Movie Row and Candy Row tasks rarely quit after initially accepting an offer (limiting the variability across participants or task experience), the participants' entry bias represented an investment with a significant impact on the cost for accepting or rejecting an offer, which had a substantial impact on the willingness of a participant to accept an offer.

The Restaurant Row and Web-Surf tasks also show promise as tools to understand how decision-making is related to addiction vulnerability and drug exposure. Results with cocaine and morphine abstinent mice have shown that these tasks have promise in the study of how drug use and cessation specifically impact decision-making (Sweis et al., 2018b). Similarly, in a version of the Web-Surf task that incorporates risky offers (Abram et al., 2019b), individuals with high trait externalizing (who thus may be at risk for addiction) were less likely than those with low trait externalizing to avoid risky offers after a loss, potentially signaling an impairment in learning from risky losses. These studies indicate the potential for experiential foraging tasks to capture decision-making processes that are relevant to human disorders, such as addiction. To further explore the potential of these tasks, we also explored the relationship of delay-discounting measures and foraging behavior to smoking status and BMI.

Using two measures of delay-discounting (the Monetary and Food Choice Questionnaires, MCQ and FCQ), we found that participants who were less willing to wait for hypothetical monetary rewards (MCQ) were also less willing to wait for hypothetical food rewards (FCQ), indicating individual differences in delay-discounting that tracked across two types of reinforcers (a primary reinforcer, food, and a secondary reinforcer, money). Similarly, we found that delay thresholds and the proportion of rewards earned on the Movie Row and Candy Row tasks were correlated, indicating a reliable individual difference in willingness to wait for rewards across two reinforcer types (movies and food, both of which are primary reinforcers). However, willingness to wait for hypothetical rewards (in the MCQ and FCQ) were not related to willingness to wait for actual rewards (in the Movie Row and Candy Row), indicating that these two measures assess different dimensions of decision-making. These findings are consistent with results obtained using a version of the Web-Surf task that incorporated risk (Abram et al., 2019b), where measures of discounting rates (based on the delay until or probability of receiving a large reward) did not relate to trait externalizing, and did not account for the relationship between externalizing and Web-Surf task performance. Our results are also consistent with findings from studies that compared foraging to forced-choice tasks (with equivalent rewards across the tasks). In these cases, behavior observed when animals are given a forced-choice between an immediate small reward and a delayed large reward can deviate strongly from behavior in the same animals when the options are presented as a stay/leave foraging decision (Stephens, 2008; Carter and Redish, 2016).

Consistent with published reports, smokers more steeply discounted hypothetical future monetary rewards compared to non-smokers. However, individuals reporting higher BMI (≥ 25) did not have higher discounting rates (k) for monetary rewards, conflicting with a previous report (Jarmolowicz et al., 2014) which found significantly higher discounting (on the MCQ) for overweight/obese compared to underweight/healthy participants. The lack of relationship between BMI and discounting in our study does fit within the larger literature on delay-discounting, with some meta-analyses in support of a BMI-discounting relationship (Amlung et al., 2016), and others finding mixed evidence (Tang et al., 2019), and more so when hypothetical rewards are used (such as in the MCQ and FCQ).

In contrast, on the foraging tasks neither smoking status nor BMI group were strongly related to delay thresholds nor to the proportion of rewards earned. And, the strongest trend observed was that smokers were, if anything, more willing to wait for rewards (with a trend toward longer delay thresholds and earning more rewards). The trend for smokers may have been driven by a tendency for smokers to report higher subjective enjoyment in the Movie Row task (in the post-video star ratings), and thus may have been more willing to wait for videos because they found them more rewarding. Such a result seems consistent with other work demonstrating that, at least for acute administration, nicotine can enhance the rewarding effects of some non-drug rewards (music and video stimuli, but not money, Perkins et al., 2017).

BMI was related to decision-making, as overweight/obese individuals were more likely to accept poor offers (higher than the participant's threshold) overall, and also showed reduced sunk-cost bias (for females, and for male smokers), as assessed by the relationship between entry bias and stay/skip decisions. Overweight/obese females not only showed less sensitivity to a measure of sunk-costs, but their decision latencies were also less sensitive to the value of an offer, suggesting that higher BMI females (BMI ≥ 25) were less impacted by the cost of obtaining a reward, and showed less deliberation when presented with difficult offers (value close to 0). While it is unknown if obesity and nicotine use are related to sunk-cost sensitivity using more traditional measures, a study by Fujino et al. (2018) found no differences in sunk-cost sensitivity in males with gambling disorders compared to male healthy controls on a task based on a scenario used in Arkes and Blumer's (1985) study of the sunk-cost effect. However, sunk-cost sensitivity in the gambling disorder sample was negatively correlated with duration of abstinence from gambling, suggesting that hypothetical decisions involving sunk-costs may be disrupted during addiction. The relationship between sensitivity to sunk-costs as assessed in foraging tasks (the Web-Surf and Movie/Candy Row) and more standard sunk-cost tasks remains to be determined, and could provide further insight into the ways in which individuals differ in their vulnerability to addictions.

Research using experiential foraging tasks such as the Restaurant Row and Web-Surf tasks provides new and important insights into decision-making. Our results with the Movie and Candy Row variants extend this work: the many cross-species behavioral similarities support the utility of these tasks to not only characterize decision-making systems, but to understand how these systems may contribute to, or be impacted by, a range of behaviors which have profound implications for public health.

## Data Availability Statement

The datasets presented in this study can be found in online repositories. The names of the repository/repositories and accession number(s) can be found at: https://osf.io/wr5un.

## Ethics Statement

The studies involving human participants were reviewed and approved by Wabash College Institutional Review Board. The patients/participants provided their written informed consent to participate in this study.

## Author Contributions

TH, KA, SA, and NS-T contributed to conception and design of the study. TH and KA collected data for in-person testing. NS-T managed online data collection, performed the statistical analysis, and wrote the first draft of the manuscript. All authors contributed to manuscript revision, read, and approved the submitted version.

## Conflict of Interest

The authors declare that the research was conducted in the absence of any commercial or financial relationships that could be construed as a potential conflict of interest.
