# Supplementary material for: Vicarious Trial-and-Error Is Enhanced During Deliberation in Human Virtual Navigation in a Translational Foraging Task
Source: Front Behav Neurosci. 2021 Apr 12;15:586159. doi: 10.3389/fnbeh.2021.586159 (PMC8072010; doi:10.3389/fnbeh.2021.586159)
Supplement: Supplementary file 1 [file Data_Sheet_1.pdf]

Supplementary Material

Vicarious trial-and-error is enhanced during deliberation in human virtual navigation in a translational neuroeconomic task

Thach Huynh, Keanan Alstatt, Samantha Abram and Neil Schmitzer-Torbert

Data

Datasets are available at https://osf.io/wr5un/.

Movie Row

A video of the current version of the Movie Row task is available at https://www.youtube.com/watch?v=q0oLFV0T728. Links to demonstration versions of the current of the Movie Row using the original Web-Surf movies (kittens, bike accidents, dancing, and landscapes) and using the second movie set of high-resolution movies (puppies, social interactions, food, and landscapes) are included with the video description, and at https://osf.io/wr5un/. This version is currently equivalent to version 4 described in these results, but lacking the fixation task.

Instructions. The instructions were adapted slightly across versions 2-4, and those included here represent version 4 of the task. Before beginning the practice phase, participants received the following instructions, divided into 1-4 sentences and presented sequentially on-screen. *In this task, you will be able to [Movie Row: watch four different kinds of short video clips, each presented on a different screen. Each movie screen plays a different type of video clip. There are videos of [Cats, Landscapes, Bike accidents, and Dancing]/[Puppies, Food, Landscapes, and Friends]]/[Candy Row (underlined here for emphasis): visit four different candy shops. Each candy shop is stocked with a different type of candy. The shops serve M&Ms, Skittles, Reese's Pieces, and [Soy nuts/Pepitas/Peanuts/Cheerios]. [Most video clips]/[At each shop, the candy] will take between 3 to 30 seconds to [load]/[be delivered]. As you approach a [movie screen]/[candy shop], a gate will lower and the [video]/[candy] type and loading time will be displayed. As you wait for [a video to load]/[the candy to be delivered], please pay*

attention to the screen and watch the blue loading bar. [If a fixation task was included: Numbers will be presented on the loading bar while the [video is loaded]/[candy is being delivered]. Pay attention to these numbers and remember the last number that is presented.] [For each video that is available]/[At each candy shop], you can either choose to [watch the video clip]/[wait for the candy to be delivered] or you can skip the [video]/[candy] and move on to the next [screen]/[shop]. [Movie Row: After watching a video, you will be asked to rate how much you liked the video on a scale of 1 to 5 stars.] To move between the [movie screens]/[candy shops], use the forward arrow key to move forward, and the left and right arrow keys to turn. We will start with a practice phase, so that you can get used to moving between the [movie screens]/[candy shops]. In the practice phase, we will ask you to [watch several videos, and skip several videos. The practice phase will end once you have watched and rated several video clips.]/[wait at each shop once, and skip each shop once.]

After completing the practice phase, participants received the following instructions before beginning the task, divided into 1-4 sentences and presented sequentially on-screen. *Congratulations, you have finished the practice phase. Now you we will start the main task, where you will choose [which videos you will watch]/[when you will wait for candy or move on to the next shop]. As you approach each [movie screen]/[shop], the [video]/[candy] type and [loading]/[delivery]time will be displayed. You can either choose to wait for the [video to load]/[candy to be delivered] or you can skip the [video]/[candy] and move on to the next [screen]/[shop]. When you are waiting for [a video to load]/[the candy to be delivered], please pay attention to the screen and watch the blue loading bar. [If a fixation task was included: Numbers will be presented on the blue bar while the video is loaded. Pay attention to these numbers and remember the last number that is presented.] At each [screen]/[candy shop], the [video]/[candy] type and [loading]/[delivery] time will be displayed. Step to the white platform if you want to wait [and watch the video]/[for the candy to be delivered]. Move on to the next [screen]/[candy shop] if you want to skip the [video]/[candy offered]. [Movie Row: Each movie screen plays a different type of video. There are videos of [Cats, Landscapes, Bike accidents, and Dancing]/[Puppies, Food, Landscapes, and Friends]]/[Candy Row: Each candy shop dispenses a different type of candy. The shops deliver M&Ms, Skittles, Reese's Pieces, and [Soy nuts/Pepitas/Peanuts/Cheerios]. [Movie Row: After watching a video, you will be asked to rate how much you liked the video on a scale of 1 to 5 stars.] The task will take approximately [X]*

minutes to complete. [Watch any video you are interested in]/[Wait for any candy that you like] or are willing to wait for. Skip any [video]/[candy offered if] you are not interested in or are not willing to wait for [Candy Row: it to be delivered]. Choosing to [watch]/[wait] or skip a [video]/[candy offer] will not change the duration of the task.

Several text instructions were also used during navigation on the task, presented at the top or bottom of the screen (except for post-video ratings and fixation test prompts). At the start of the practice phase, when participants were initially placed on the virtual track: *Please move straight ahead to the first [screen]/[shop]*. In the practice phase, participants were instructed to accept one offer at each reward zone: *Please [watch this video clip. To load this video]/[wait for the candy at this shop. To deliver the candy], please stand on the white platform and look at the [screen]/[candy dispenser]*. After reaching the platform: *Please turn to look at the [screen]/[candy dispenser]*. During the delay: *Wait for the [video to load]/[candy to be delivered]*. Participants were also instructed to skip one offer at each reward zone in the practice phase: *Please skip this [video clip]/[shop] and move to the next screen]/[shop]*.

When entering an offer zone, the offer was presented at the bottom of the screen. In the practice phase, the phrasing was: *Here you can [watch a]/[get] [reward type] [Movie Row: video], [line break] which will [load]/[be delivered] in [X] seconds*. In the test phase, the phrasing was shortened to: *[reward type][Movie Row: video][line break] [X] seconds [to load]/[until delivery]*.

After stepping to the platform in the test phase: *Please turn to look at the [screen]/[candy dispenser], or move on to the next [movie screen]/[candy shop]*. After reaching the platform: *Wait for the [video to load]/[candy to be delivered], or turn and move on to the next [screen]/[shop]*. If the delay was completed, and if no fixation task was used, a prompt was displayed to allowing them to start reward delivery: *Click [Play]/[Start] or press Enter to start [the video]/[delivering the candy]*. If a fixation task was used, the instructions for the fixation test were: *What was the last number presented while the [video was being loaded]/[delivery was being prepared]? Please click the correct option.*

After video clips were completed in the Movie Row task, post-video rating instructions for the practice phase were: *How well did you like this video clip? Click a star to rate the video. (1 star = did not like the video, 5 stars = extremely liked video) Click Next once you have*

finished rating the video. In the test phase, these instructions were shortened: *How well did you like this video clip? Click a star to rate the video (1 to 5 stars). Click Next to move on.*

After completing reward delivery, or upon re-entering the offer zone after making a skip decision: *Please move on to the next screen*. If the participant attempted to travel counterclockwise, and reached a fence blocking the path behind them: *Wrong direction, please turn around and move on the next movie screen.*

Recruitment and compensation

Undergraduates received course credit, extra credit or coupons for a campus coffee shop ($10) for participation, while mTurk workers were paid between $8-$8.50 USD for completing one version of the Movie Row task, and those recruited for the second sample also received $0.15 USD for completing a demographics survey, and $2.50 USD for completing a screening questionnaire. Participation by mTurk workers was limited to individuals in the United States, and those who had an approval rate for previous assignments of at least 90%. Community members were recruited by direct mail, online advertisement (Facebook, Craigslist) and public online classified ads posted on the Wabash College website, and received a $6 gift card for completing an initial survey battery, and $25 for travelling to campus and completing both the Movie and Candy Row tasks.

Participants by version

Data for the studies reported here were collected in the context of several undergraduate research projects, conducted as course research projects, summer research internships, independent study, and senior capstone research. Across these research studies, the Monetary Choice questionnaire (using the values and delays, and a related version, Wang, Reed, Baugh, & Fercho, 2018) was administered to most participants, as well as information used to calculate BMI (height and weight). A subset of participants completed measures assessing nicotine use, and individuals who stated that they used cigarettes or e-cigarettes (e.g. vaping) were classified as “smokers” while participants who indicated no use of any nicotine products were classified as “non-smokers.” Most samples (except undergraduates participating in a study of the effects of glucose-consumption on decision-making) completed screening surveys or follow-up surveys that included additional measures as part of screening questionnaires or follow-up surveys,

including measures of dieting attempts, dieting intentions, measures of trait mindfulness, histories of drug use, exercise, and other measures not reported here. Undergraduates were recruited from a private college with an all-male student body, typically between the ages of 18-22, while online recruitment targeted equal recruitment of females and males ages 18 years and older.

Version 1. Male undergraduates (n = 30), were recruited for a study of the effect of glucose ingestion on decision-making. Participants were asked to fast for at least four hours before the study, and were tested at 8am or 4:30pm each day. Before completing the task, participants provided a blood glucose measurement, completed a version of the Monetary Choice Questionnaire, consumed a drink (water, glucose [26.2g in 12 oz], water after rinsing with 6 oz of the glucose solution), waiting a total of 13 minutes for absorption, and then completing a second blood glucose measurement and an alternate version of the MCQ. Participants then completed the Movie Row task, tested on PCs divided by partitions. Headphones were provided, so that participants could hear the audio presented with each video.

Version 2. Male undergraduates (n = 10) and workers from Amazon’s Mechanical Turk (mTurk) service (n = 50, 29 females) were recruited to complete the Movie Row task administered online. Participants recruited from mTurk had previously participated in an unrelated virtual navigation study, and completed a screening survey as part of that study. Participants completed a version of the MCQ as part of the screening questionnaire or immediately before being directed to the Movie Row task.

Version 3. Male undergraduates (n = 21) and mTurk workers (n = 97, 51 females, 45 males, 1 non-binary). Undergraduates were tested as a second sample in the glucose study described for version 1 above. Participants from mTurk were recruited for a project examining the relationship of smoking status to delay discounting and performance on the Movie Row task. Participants who reported use of other drugs (besides nicotine or alcohol) in the past 30 days were excluded, as were those who indicated drinking more than 9 times in the previous 30 days, or any alcohol consumption if they also indicated any history of alcohol abuse. From an initial sample of 450 participants who completed the screening questionnaire, 51 smokers and 237 non-smokers were eligible and recruited for the Movie Row task, and a total of 97 participants (20 smokers) completed the Movie Row task.

Version 4 (Movie Row and Candy Row). Male undergraduates (n = 83) and members of the local community (n = 34, 19 females, 15 males) completed the Movie Row task. Male undergraduates (n = 83) and members of the local community (n = 34, 19 females, 15 males) completed the Movie Row task. The undergraduates were recruited for a study of the relationship between decision-making and dieting success, and completed a screening questionnaire that assessed BMI, the MCQ, and the Food Choice Questionnaire (Hendrickson, Rasmussen, & Lawyer, 2015). The local community members were recruited through mailings and online advertisement to complete the online screening questionnaire, which assessed BMI and smoking status, and a total of 137 complete responses were received (73 females). Participants were not recruited for the Movie Row task if they reported indicated drinking more than 9 times in the previous 30 days, or any alcohol consumption if they also indicated any history of alcohol abuse. A total of 58 participants from the local community were recruited to be tested in-person on the Movie and Candy Row tasks (n = 16 non-smokers with BMI < 25, n = 21 non-smokers with BMI > 25, and n = 20 smokers (8 with BMI < 25)).

Thirty-one of the male undergraduates completed one session of the Movie Row task online (using the alternate videos, with puppies, social interactions, food, and landscapes). Sixty-nine of the participants also completed the Candy Row task in addition to at least one Movie Row session.

Delay discounting surveys

Responses for both the Monetary Choice Questionnaire and Food Choice Questionnaire monetary and food versions of the survey were orderly, judged by the percentage of responses for each participant which were consistent with their estimated discounting rate (k). The mean percentage of responses consistent with estimated k values for the MCQ (for small (98%), medium (99%) and large (99%) reward magnitudes) were slightly higher than for the FCQ (for small (93%), medium (94%) and large (94%) reward magnitudes), but were both consistent with those reported by Hendrickson, Rasmussen and Lawyer (2015).

Gender differences. An ANOVA was used to analyze discounting rates ($\log_{10}$ k) for the MCQ with Magnitude (small, medium, large) as a within-subjects factor, and Gender (male, female) as a between-subjects factor. Discounting rates decreased as the reward size increased ($F(2, 672) = 750$, $p < 0.001$, $\eta_p^2 = 0.69$), and were not related to Gender (Gender: ($F(1, 336) < 1$,

n.s., $\eta_p^2 < 0.001$, Gender × Magnitude: F(2, 672) = < 1, n.s., $\eta_p^2 < 0.001$). For the subset of participants who completed the FCQ, the main effect of Magnitude did not reach significance (F(2, 338) = 2.8, p = 0.063, $\eta_p^2 = 0.016$), which was qualified by a significant Gender × Magnitude interaction (F(2, 338) = 7.7, p < 0.001, $\eta_p^2 = 0.044$), while the main effect of Gender was not significant (F(1, 169) = 2.4, p = 0.13, $\eta_p^2 = 0.014$). Post-hoc tests (Bonferroni corrected, $\alpha = 0.05$) revealed that for the FCQ, discount rates for males for small magnitude rewards were significantly higher than for medium and large rewards, but for females there were no differences across the three reward magnitudes.

Row tasks

Stated preferences across video types. Participants provided self-report measures of their stated reward preferences (the ratings given immediately after watching each video or the pre-task ratings of each food, and the post-task rankings of the video/food types from most to least favorite). Additionally, in the Candy Row task another measure of reward preference was the frequency of sampling the reward magazine (to retrieve earned rewards). Abram et al. (2016) reported a significant interaction between gender and stated preferences for the four movie types in the Web-Surf task, and similar results were obtained for the Movie Row (see Supplementary Figure 1A). For the videos from the original Web-Surf task, using a two-factor ANOVA with Gender (female, male) as a between-subjects factor and Reward Type (Kitten, Accident, Landscape and Dance) as a within-subjects factor, there was a main effect of Reward Type (F(3, 855) = 83.9, p < 0.001, $\eta_p^2 = 0.23$), and no main effect of Gender (F(1, 285) = 1.7, p = 0.19, η_p^2 = 0.006). As predicted, the interaction of Reward Type × Gender was significant (F(3, 855) = 133.0, p < 0.001, $\eta_p^2 = 0.10$), with females giving higher ratings to Kitten videos than did males, and males giving higher ratings to Landscape videos than did females. Average post-video ratings did not differ significantly by gender for Accident and Dance videos.

In a separate ANOVA for the second set of movies (Supplementary Figure 1B), there was a main effect of Reward Type (Puppies, Social, Food and Landscape, $F(3, 105) = 15.6$, $p < 0.001$, $\eta_p^2 = 0.31$), and no main effects of Gender ($F(1, 35) < 1$, n.s., $\eta_p^2 = 0.02$) nor an interaction of Reward Type × Gender ($F(3, 105) < 1$, n.s., $\eta_p^2 = 0.008$). Similar results were found for the Candy Row task (Supplementary Figure 1C-D) for pre-task enjoyment ratings given to the food options, where there was a main effect of Reward Type (M & Ms, Reese's Pieces, Skittles and Other, $F(3, 192) = 6.2$, $p < 0.001$, $\eta_p^2 = 0.09$), and no main effects of Gender ($F(1, 64) < 1$, n.s., $\eta_p^2 = 0.008$) nor an interaction of Reward Type × Gender ($F(3, 192) < 1$, n.s., $\eta_p^2 = 0.01$). For magazine entries, there was also a main effect of Reward Type ($F(3, 171) = 3.6$, $p = 0.015$, $\eta_p^2 = 0.06$), while the Type × Gender interaction was not significant ($F(3, 171) < 1$, n.s., $\eta_p^2 = 0.008$). Males did make more magazine entries on average than females ($F(1, 57) = 4.8$, $p = 0.032$, $\eta_p^2 = 0.08$) across the four reward types, though neither main effect nor the interaction were significant when the all-male undergraduate sample was excluded and the analysis was conducted for 33 individuals recruited from the local community (ps > 0.1, η_p^2s < 0.09).

Overriding preferences

Sweis and colleagues (2018) have reported that time spent in the offer zone and VTE are increased when mice skip offers for their most preferred food, and that increased VTE is associated with improved decision-making (associated with an increased probability of skipping low-value offers, which are above the threshold for the restaurant). To determine if similar patterns were obtained for humans, we examined decision latency and offer zone behaviors when participants made decisions (stay/skip) which were consistent or inconsistent with their preferences.

In contrast to results on the Restaurant Row with mice, humans tested on the Movie/Candy Row tasks did not show increased decision times when skipping offers for their most-preferred rewards (Supplementary Figure 4). In an ANOVA with post-task Rank (1-4) and Decision (skip/stay) as within-subjects factors, and Gender (female/male) as a between-subjects factor, there was no evidence for a Rank × Decision interaction ($F(3, 483) < 1$, n.s., $\eta_p^2 = 0.003$) for decision latencies (z-scored). There was a significant main effect of Rank ($F(3, 483) = 2.9$, $p = 0.034$, $\eta_p^2 = 0.018$), with participants making faster choices in their most-preferred reward location. There was a significant Gender × Decision interaction ($F(1, 161) = 9.5$, $p = 0.003$, $\eta_p^2 = 0.056$), where males had longer decision latencies when skipping offers, and females having longer decision latencies when accepting offers. No other main effects or interactions were significant ($p > 0.19$).

While we did not find that skip decisions were significantly slower for preferred rewards, decision latencies were elevated on trials in which participants violated their delay thresholds. Decision latencies (z-scored) were higher when accepting low-value offers (above threshold) or rejecting high-value offers (below threshold, see Supplementary Figure 5A-B). Analyzing decision latencies (z-scored) using an ANOVA with Gender as a between-subjects factor, and Decision (skip/stay) and Value (< threshold, > threshold) as within-subjects factor, there was a significant Value × Decision interaction ($F(1, 211) = 32.9$, $p < 0.001$, $\eta_p^2 = 0.13$). As with the analysis by ranking, there was a significant Gender × Decision interaction ($F(1, 211) = 8.5$, $p < 0.001$, $\eta_p^2 = 0.039$), and the Gender × Value × Decision interaction approached significance ($F(1, 211) = 3.9$, $p = 0.05$, $\eta_p^2 = 0.018$). Examining females and males with separate ANOVAs, there was a significant Value × Decision interaction for both genders ($p < 0.03$), and the main difference observed was that females did not show increased latencies when accepting low-

quality offers. This gender difference may indicate a possible confound with age, and when the ANOVA was restricted to participants who were 40 years and younger, the Gender × Value × Decision interaction was no longer significant ($p = 0.28$), while the significant Value × Decision interaction remained ($p < 0.001$).

These results contrast with results in mice (Sweis et al., 2018, see Supplementary Material), where choices were fastest when accepting offers in general (both above and below threshold), indicating a possible species difference. When successfully rejecting offers above threshold, mice were slower to make a decision and engaged in more VTE, suggesting a specific role for deliberation in rejecting low-quality offers for preferred rewards. In contrast, humans tested here in the virtual version of the task made their fastest choices when acting consistently with their preferences (accepting high-value offers, rejecting low-value offers), and were slower when acting inconsistently with these preferences.

Several other behavioral measures were also sensitive to these delay threshold violations (see Supplementary Figure 5C, E-H), and were elevated when participants made decisions that were not consistent with their delay thresholds. In ANOVAs conducted for each behavioral measure, there were significant Value × Decision interactions for total distance travelled (z-scored, $F(1, 211) = 10.3$, $p = 0.002$, η_p^2 = 0.0.047), total rotation (mean-centered, $F(1, 211) = 10.4$, $p = 0.002$, $\eta_p^2 = 0.047$), rotation reversals ($F(1, 210) = 13.5$, $p < 0.001$, $\eta_p^2 = 0.06$), pausing (z-scored, $F(1, 211) = 19.3$, $p < 0.001$, $\eta_p^2 = 0.084$), as well as reaction time ($F(1,170) = 4.7$, $p = 0.031$, $\eta_p^2 = 0.027$). The results for total rotation and rotation reversals were qualified by significant Gender × Value × Decision interactions (total rotation: $F(1, 211) = 10.5$, $p = 0.001$, $\eta_p^2 = 0.047$, reversals: $F(1, 210) = 12.2$, $p < 0.001$, η_p^2 =0.055), and separate ANOVAs by gender indicated that Value × Decision interactions were significant in males (ps < 0.001), but not in

females (ps > 0.85), and this pattern of results was not changed by restricting the analysis to participants 40 years and younger, or by omitting the all-male undergraduate sample.

These results indicate that when participants made decisions inconsistent with their delay thresholds, their latency to make a choice was lengthened, during which they travelled farther and tended to pause for a longer duration. Males were also more likely to change their direction of rotation, and rotate more overall before completing their choice on these trials.

Sequential choices

As described in the main text, participants had slower latencies on Stay/Skip trials (skipping an offer after accepting the previous offer), consistent with results reported for the Web-Surf (Abram, Redish, & MacDonald III, 2019). On Stay/Skip trials, participants also travelled farther on average, paused longer, and were more likely to reverse their direction of rotation (Supplementary Figure 5) compared to Skip/Skip trials. In separate ANOVAs for other behavioral measures, there were significant $\text{Decision}_{(i)} \times \text{Decision}_{(i-1)}$ interactions for the distance travelled (z-scored, $F(1, 242) = 23.3$, $p < 0.001$, $\eta_p^2 = 0.014$), pausing ($F(1, 238) = 8.0$, $p = 0.005$, $\eta_p^2 = 0.007$), and rotation reversals ($F(1, 242) = 44.1$, $p < 0.001$, $\eta_p^2 = 0.015$). The pattern for total rotation (Figure 6D) was similar to that of distance travelled, but the interaction was not significant (total rotation: $F(1, 242) = 2.8$, $p = 0.39$, $\eta_p^2 < 0.001$). Participants spent more time paused on Stay/Skip trials compared to Skip/Skip trials ($t(239) = 5.0$, $p < 0.001$), but overall participants spent more time paused if they accepted the previous offer (main effect of $\text{Decision}_{(i)}$: $F(1, 238) = 35.6$, $p < 0.001$, $\eta_p^2 = 0.054$). Similarly, participants travelled shorter distances ($F(1, 239) = 55.4$, $p < 0.001$, $\eta_p^2 = 0.11$), and rotated less overall $F(1, 238) = 12.9$, $p < 0.001$, $\eta_p^2 = 0.027$) when accepting offers, but these effects were consistent with the overall bias

for participants to enter the left-hand side of the offer zone (entry bias: M = -12.6%, SD = 19.3%). No main effects or interactions were significant for reaction time (z-scored, ps > 0.14, $\eta_p^2 < 0.0025$), except that reaction times were slower if participants had accepted the previous offer (main effect of $\text{Decision}_{(i-1)}$: $F(1, 238) = 5.7$, $p = 0.018$, $\eta_p^2 = 0.009$). Except for the initial ANOVA for decision latencies, none of the Gender × $\text{Decision}_{(i)}$ × $\text{Decision}_{(i-1)}$ interactions were significant (ps > 0.10, $\eta_p^2 < 0.0016$).

References

Abram, S. V., Breton, Y., Schmidt, B., Redish, A. D., & MacDonald III, A. W. (2016). The web-surf task: A translational model of human decision-making. *Cognitive, Affective & Behavioral Neuroscience, 16*(1), 37-50. doi:10.3758/s13415-015-0379-y

Abram, S. V., Redish, A. D., & MacDonald III, A. W. (2019). Learning from loss after risk: Dissociating reward pursuit and reward valuation in a naturalistic foraging task. *Frontiers in Psychiatry, 10*, 359. doi:10.3389/fpsyt.2019.00359

Hendrickson, K. L., Rasmussen, E. B., & Lawyer, S. R. (2015). Measurement and validation of measures for impulsive food choice across obese and healthy-weight individuals. *Appetite, 90*, 254-263. doi:10.1016/j.appet.2015.03.015 [doi]

Sweis, B. M., Redish, A. D., & Thomas, M. J. (2018). Prolonged abstinence from cocaine or morphine disrupts separable valuations during decision conflict. *Nature Communications, 9*(1), 2521. doi:10.1038/s41467-018-04967-2

Wang, X. T., Reed, R. N., Baugh, L. A., & Fercho, K. A. (2018). Resource forecasting: Differential effects of glucose taste and ingestion on delay discounting and self-control. *Appetite, 121*, 101-110. doi:10.1016/j.appet.2017.11.083

Supplementary Table 1.

Description of samples tested with each Movie Row version.

Version	Location	Source	Videos	n	Age
1	Lab	Undergraduate	original	30 (0 females)	
2	Online	Undergraduate	original	10 (0 females)	
	Online	mTurk	original	50 (29 females)	43.3 (6.5)
3	Lab	Undergraduate	original	21 (0 females)	
	Online	mTurk	original	97 (51 females, 1 non-binary)	36.4 (10.2)
4	Lab	Undergraduate	original	57 (0 females)	19.1 (1.2)
	Online	Undergraduate	new	30 (0 females)	18.9 (1.1)
	Lab	Community	new	34 (19 females)	46.4 (14.1)

Age and other demographics were not collected for two of the undergraduate samples, but ages were likely to have fallen in the 18-22 age range. Participants were tested with the original Web-Surf videos (kittens, bike accidents, dancing, and landscapes) or a newer, higher-resolution set (puppies, social interactions, food, and landscapes). For version 4, four undergraduate participants completed two versions of the Movie Row task (with different sets of movie clips), with one session completed in the laboratory, and one completed online.

Supplementary Table 2.

Description of samples by smoking status or BMI group, separated by task and reward type.

Task	Videos	Group	n	Age
Movie Row	original	Non-smokers	111 (62 females)	38.4 (9.9)
		Smokers	34 (18 females)	39.9 (9.1)
Movie Row	new	Non-smokers	26 (14 females)	48.4 (14.7)
		Smokers	8 (5 females)	40.0 (9.7)
Candy Row	-	Non-smokers	26 (13 females)	48.0 (14.2)
		Smokers	7 (5 females)	41.4 (9.5)
Movie Row	original	Underweight/Healthy	88 (34 females)	32.0 (12.3)
		Overweight/Obese	105 (40 females)	33.6 (12.0)
Movie Row	new	Underweight/Healthy	17 (9 females)	37.2 (18.6)
		Overweight/Obese	27 (10 females)	42.0 (16.0)
Candy Row	-	Underweight/Healthy	28 (9 females)	30.2 (17.0)
		Overweight/Obese	40 (9 females)	34.2 (16.5)

Self-reported smoking status and height/weight (used to calculate BMI) were available from a subset of participants tested on the Movie and Candy Row tasks. Smoking status: Non-smokers – reported no current use of nicotine products, Smokers – reported current use of cigarettes or e-cigarettes (vaping). BMI: Healthy/Underweight – BMI < 25.0, Overweight/Obese – BMI $\geq$ 25.0.

Supplementary Table 3.

Value models for latency, rotation, distance and pausing

	β	95% CI	p	p-adj
Decision latency (z-scored)				
|value|	-0.010	[-0.012, -0.008]	**<0.001**	**0.002**
value type	-0.012	[-0.059, 0.041]	0.64	0.72
gender	-0.052	[-0.10, -0.001]	**0.046**	0.064
|value|:value type	-0.0006	[-0.005, 0.003]	0.80	0.80
|value|:gender	0.006	[0.001, 0.010]	**0.014**	**0.025**
value type:gender	0.15	[0.088, 0.21]	**<0.001**	**0.002**
Total rotation (z-scored)				
|value|	-0.011	[-0.013, -0.009]	**<0.001**	**0.002**
value type	0.020	[-0.027, 0.073]	0.44	0.44
|value|:value type	-0.0044	[-0.0087, -0.0003]	**0.050**	0.067
Rotation reversals				
|value|	-0.0076	[-0.0092, -0.0059]	**<0.001**	**0.002**
value type	0.016	[-0.021, 0.053]	0.42	0.42
|value|:value type	-0.0042	[-.0075, -0.0014]	**0.006**	**0.008**
Distance (z-scored)				
|value|	-0.0073	[-0.0094, -0.0053]	**<0.001**	**0.001**
value type	-0.11	[-0.15, -0.05]	**<0.001**	**0.001**
|value|:value type	-0.0022	[-0.0083, 0.0048]	0.50	0.50
Pausing (z-scored)				
|value|	-0.0041	[-0.0062, -0.0020]	**<0.001**	**0.002**
value type	0.027	[-0.023, 0.081]	0.32	0.42
|value|:value type	0.0009	[-0.0031, 0.0056]	0.65	0.65
Reaction time (z-scored)				
|value|	-0.0041	[-0.0065, -0.0016]	**<0.001**	**0.004**
value type	-0.021	[-0.076, 0.035]	0.46	0.46
|value|:value type	0.0030	[-0.0016, 0.0080]	0.23	0.30

Supplementary Table 3 (continued).

Behavioral measures were regressed separately onto absolute value of the offer (|value|), value type (-0.50 = < threshold, +0.50 = > threshold) and the *|value|:value type* interaction (*y ~ |value| + value type + |value|:value type*). Model for decision latencies (described in the main text) includes gender (-0.50 = male, +0.50 = female) and interactions of |value| and value type with gender (*y ~ |value| + value type + gender + |value|:value type + |value|:gender + value type:gender*). Bold indicates $p < 0.05$.

Supplementary Table 4.

Value models by smoking status and BMI group

	β	95% CI	p	p-adj
Decision latency (z-scored)				
Smoking status				
|value|	-0.011	[-0.014, -0.0077]	**<0.001**	**0.004**
value type	-0.045	[-0.11, 0.026]	0.19	0.45
smoker	0.16	[-0.057, 0.093]	0.69	0.69
|value|:value type	0.0023	[-0.0028, 0.0078]	0.41	0.57
|value|:smoker	-0.002	[-0.008, 0.0038]	0.53	0.61
value type:smoker	0.047	[-0.029, 0.14]	0.28	0.48
BMI group				
BMI < 25				
|value|	-0.0091	[-0.013, -0.006]	**<0.001**	**0.002**
value type	-0.13	[-0.21, -0.037]	**0.006**	**0.008**
gender	0.0046	[-0.090, 0.083]	0.91	0.91
|value|:value type	0.0094	[0.0031, 0.017]	**0.006**	**0.008**
|value|:gender	-0.0013	[-0.0085, 0.0054]	0.75	0.87
value type:gender	0.16	[0.054, 0.24]	**<0.001**	**0.002**
BMI ≥ 25				
|value|	-0.0092	[-0.013, -0.0057]	**<0.001**	**0.004**
value type	0.062	[-0.026, 0.14]	0.13	0.13
gender	-0.11	[-0.18, -0.019]	**0.012**	**0.017**
|value|:value type	-0.0063	[-0.013, 0.0004]	0.076	0.089
|value|:gender	0.010	[0.0042, 0.017]	**0.004**	**0.009**
value type:gender	0.12	[0.027, 0.21]	**0.010**	**0.017**

Decision latency (log_{10} transformed and z-scored) was regressed separately onto absolute value of the offer (|value|), value type (-0.50 = < threshold, +0.50 = > threshold) for smokers (-0.50 = non-smoker, +0.50 = smoker) and by BMI group. All 2-way interactions were included in the model. Bold indicates $p < 0.05$.

Supplementary Table 5.

Relationship between entry bias and stay/skip decisions

	β	95% CI	p	p-adj
Choice (skip/stay)				
value type	0.63	[0.61, 0.64]	**<0.001**	**0.002**
entry bias	-0.16	[-0.19, -0.14]	**<0.001**	**0.002**
gender	-0.004	[-0.028, 0.019]	0.75	0.75
value type:entry bias	0.060	[0.015, 0.11]	**0.020**	**0.028**
value type:gender	-0.008	[-0.029, 0.014]	0.48	0.56
entry bias:gender	0.083	[0.027, 0.13]	**<0.001**	**0.004**
BMI group				
Females				
value type	0.62	[0.60, 0.64]	**<0.001**	**0.002**
entry bias	-0.13	[-0.18, -0.090]	**<0.001**	**0.002**
bmi group	0.022	[-0.014, 0.067]	0.29	0.29
value type:entry bias	0.065	[-0.021, 0.15]	0.14	0.16
value type:bmi group	-0.048	[-0.080, -0.015]	**<0.001**	**0.002**
entry bias:bmi group	0.15	[0.054, 0.24]	**0.002**	**0.003**
Males				
value type	0.63	[0.62, 0.65]	**<0.001**	**0.002**
entry bias	-0.21	[-0.26, -0.17]	**<0.001**	**0.002**
bmi group	-0.026	[-0.007, 0.058]	0.14	0.19
value type:entry bias	0.048	[-0.026, 0.12]	0.19	0.23
value type:bmi group	-0.061	[-0.088, -0.036]	**<0.001**	**0.002**
entry bias:bmi group	0.026	[-0.051, 0.12]	0.53	0.53

Supplementary Table 5 (continued).

	β	95% CI	p	p-adj
Smoking status				
Females				
value type	0.62	[0.60, 0.64]	**<0.001**	**0.002**
entry bias	-0.13	[-0.19, -0.078]	**<0.001**	**0.002**
smoker	-0.003	[-0.056, 0.044]	0.87	0.87
value type:entry bias	0.094	[0.016, 0.19]	**0.030**	0.053
value type:smoker	-0.013	[-0.050, 0.027]	0.50	0.58
entry bias:smoker	-0.043	[-0.14, 0.071]	0.46	0.58
Males				
value type	0.60	[0.58, 0.63]	**<0.001**	**0.002**
entry bias	-0.18	[-0.26, -0.11]	**<0.001**	**0.002**
smoker	0.038	[-0.024, 0.10]	0.24	0.34
value type:entry bias	0.002	[-0.12, 0.11]	0.97	0.97
value type:smoker	-0.008	[-0.052, 0.039]	0.72	0.84
entry bias:smoker	0.21	[0.052, 0.35]	**0.008**	**0.014**

Decisions (0 = skip, 1 = stay) were regressed onto value type of the offer (0 = < threshold, 1 = > threshold), entry bias (-0.50 to +0.50 of the hallway width, 0 = hallway center) and the *|value|:value type* interaction (*y ~ value type + entry bias + value type:entry bias*). In separate regressions, smoking status (0 = non-smoker, 1 = smoker) or BMI group (1 = BMI < 25, 2 = BMI ≥ 25) were added to the model along with all interactions between the three terms. Bold indicates p < 0.05.

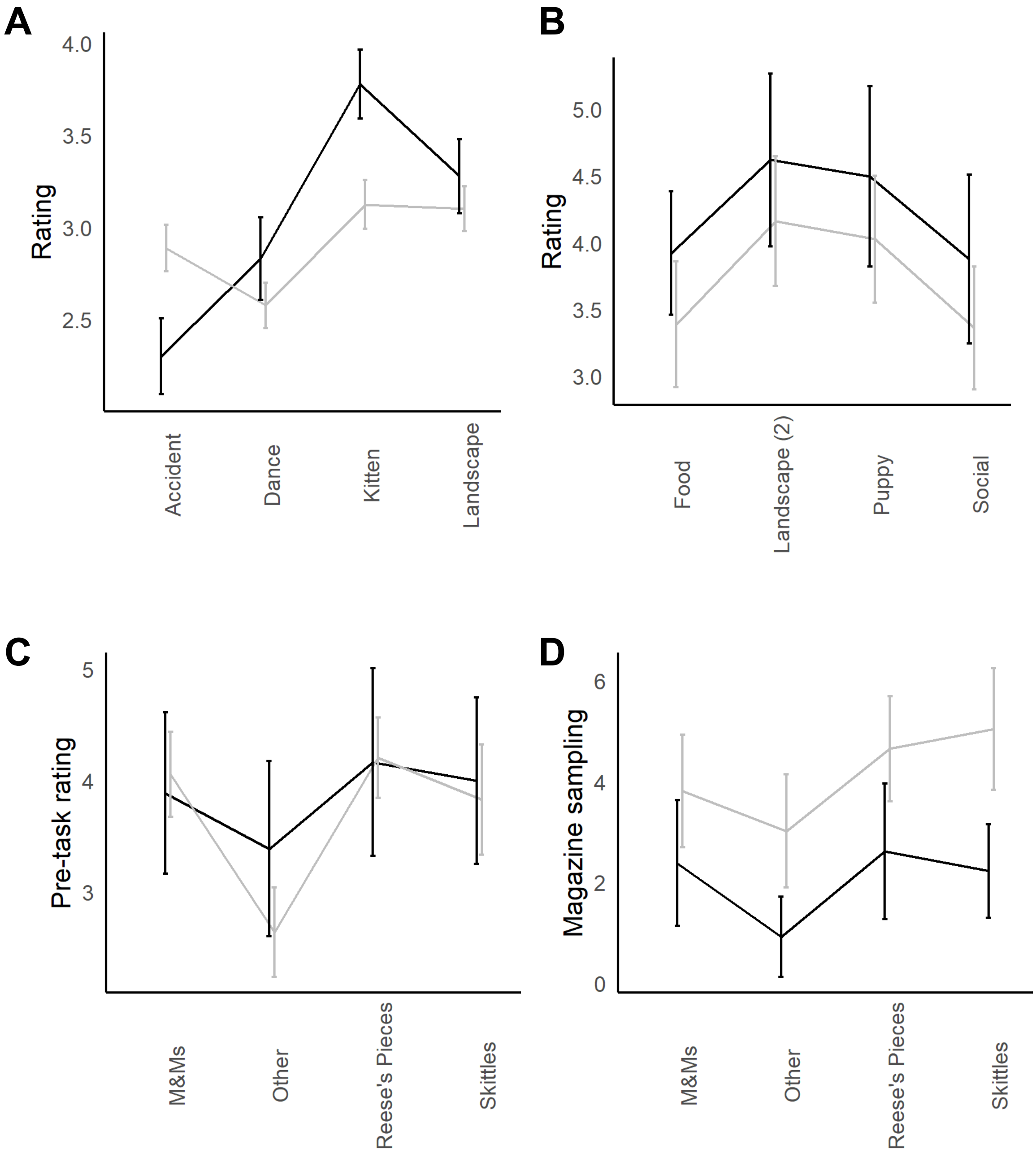

Supplementary Figure 1. Reward preferences by gender. A-B: Post-video ratings (1-5 stars) for the Movie Row task using the original Web-Surf (A) or a second (B) set of movies. C-D: Candy Row task. C: Pre-task ratings of enjoyment each reward (1-6). D: Number of magazine entries per trial for each reward type. Gender differences were only significant for the original Web-Surf videos (A), with males rating Accident videos more highly, and females rating Kitten videos more highly.

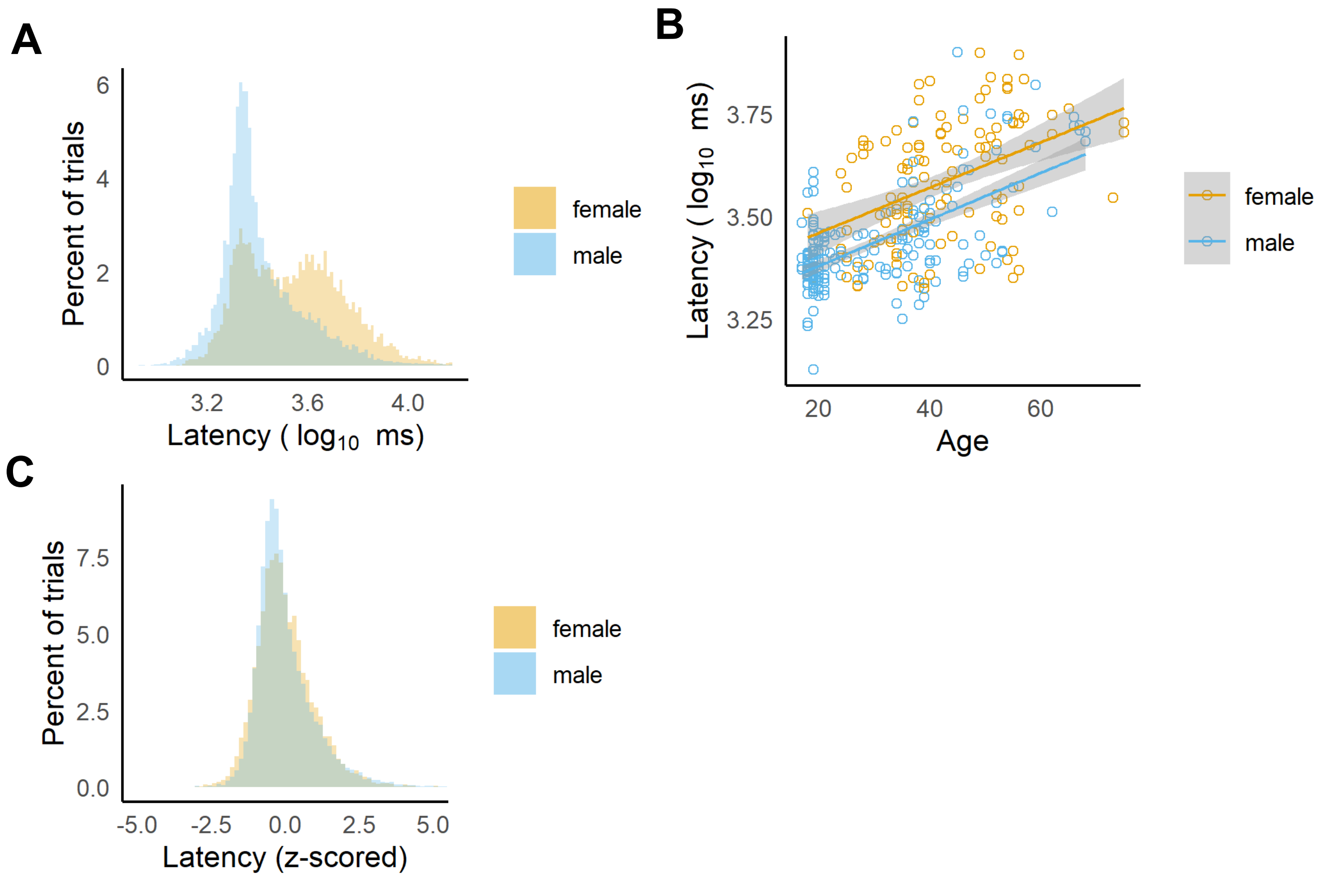

Supplementary Figure 2. Decision latencies. *A*: After $\log_{10}$ transformation, the distribution of decision latencies (across all trials from the first session for all participants) was bimodal for females and had a strong positive skew for males. *B*: Average decision latency ($\log_{10}$ milliseconds) was related to age and gender, with females having longer latencies on average, and latencies increasing with age. *C*: After z-scoring the $\log_{10}$ transformed decision latencies (within session), the differences between females and males was reduced, but males tended to have a stronger positive skew in their decision latencies.

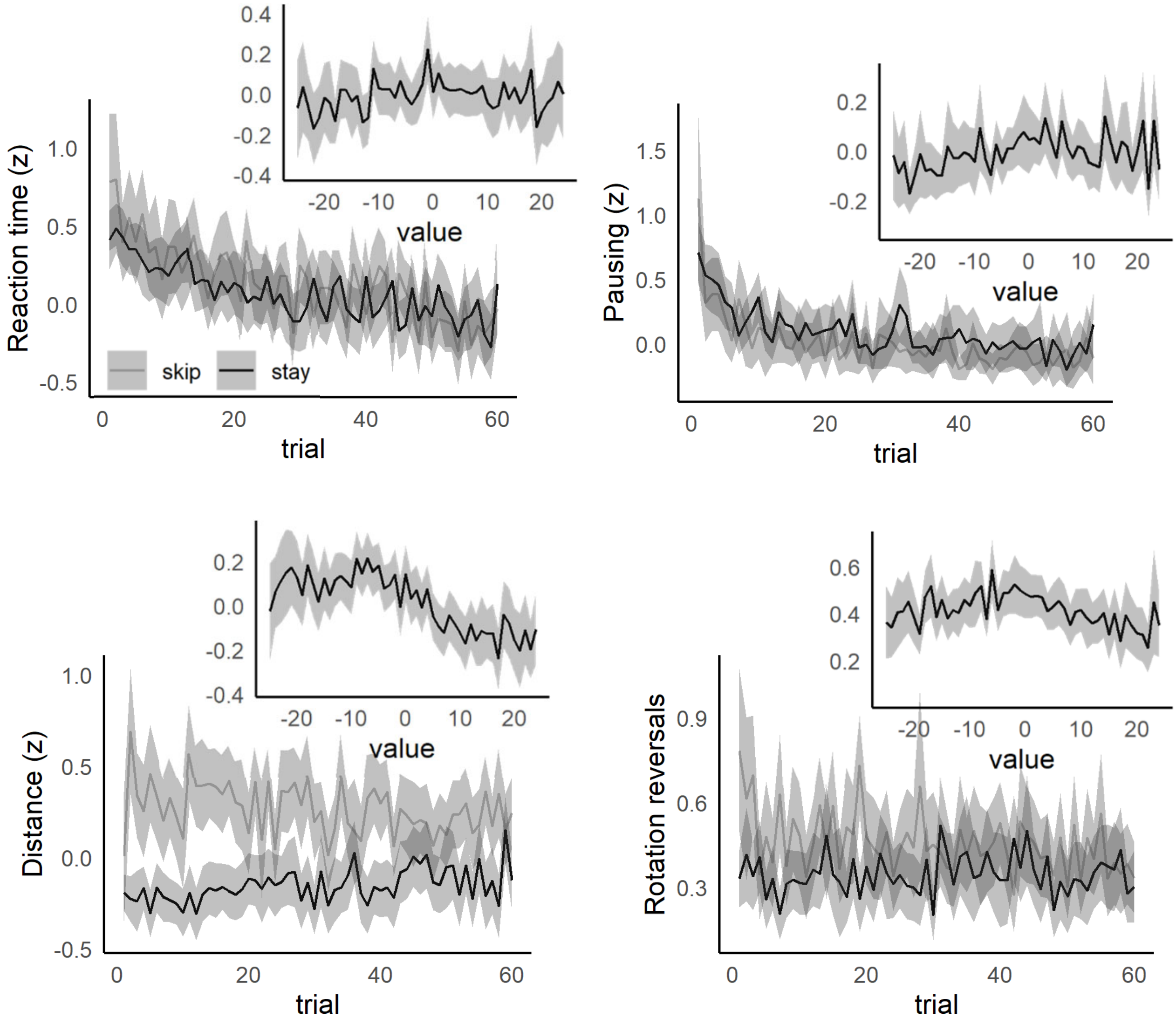

Supplementary Figure 3. Cross-session changes in behavior and relationship to deliberation. Across the session, reaction time and time spent paused decreased for both stay and skip decisions. For rotation reversals and distance travelled, a similar decrease across trials was observed for skip decisions. For each measure, plots relative to trial were calculated across participants, plots relative to value were calculated by binning values at 1 second intervals between -25 to +25 seconds, then averaging within participant before calculating means and confidence intervals. Lines indicate means, shaded areas indicate 95% confidence intervals.

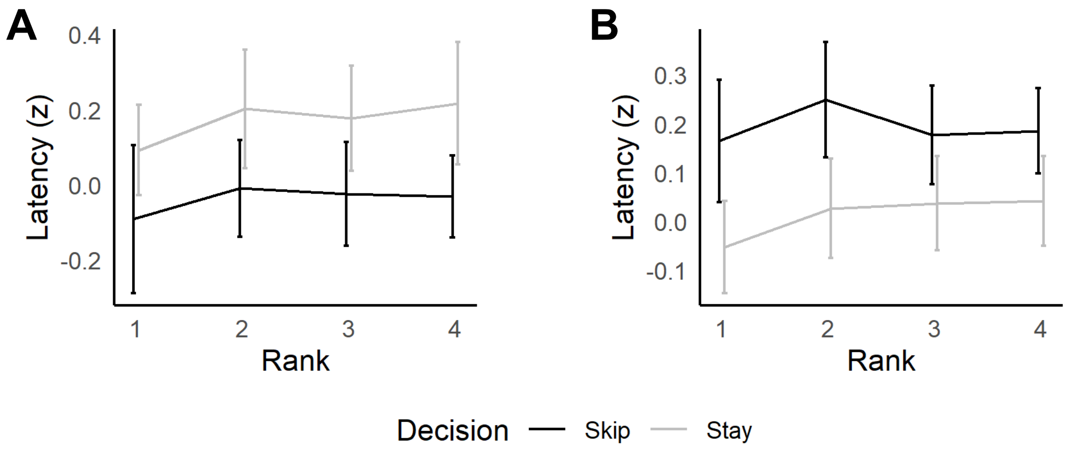

Supplementary Figure 4. Decision latency by post-task ranking and gender. Decision latencies were slower for stay decisions for females (*A*) and skip decisions for males (*B*). Overall, latencies were also faster for the favorite rewards (ranked 1). No significant interactions with rank were observed.

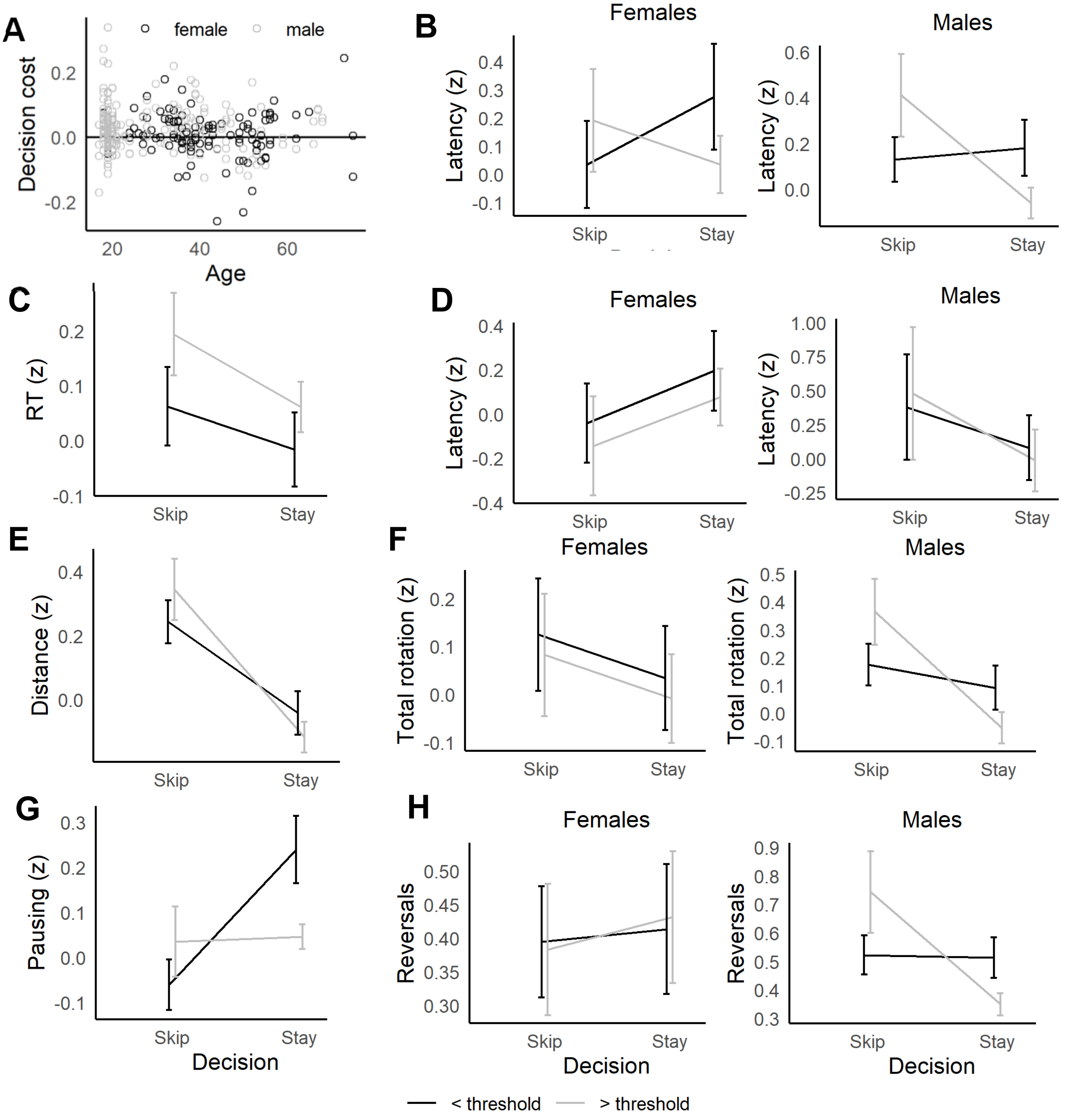

Supplementary Figure 5. Behavior when making choices inconsistent with delay thresholds. *A*: Average difference between inconsistent and consistent choices (Decision cost) versus age. Overall, 69% of males versus 56% of females had a positive decision cost (and had longer latencies when accepting offers above threshold versus below, or skipping offers below threshold versus above). This gender difference was related to age, with more participants 40 years old and younger having decision costs over 0 (71% of males, 59% of females, panel *B*) than those who were over 40 years of age (56% of males, 53% of females, panel *D*). *C*: Reaction times were longer when participants skipped good offers. *E-H*: Participants travelled farther (*E*) and paused longer (*G*) when accepting offers above threshold and skipping offers below threshold. *F & H*: A similar pattern was seen for the total amount of rotation (*F*) and number of reversals in rotation direction (*H*) in the offer zone, but only for males (right).

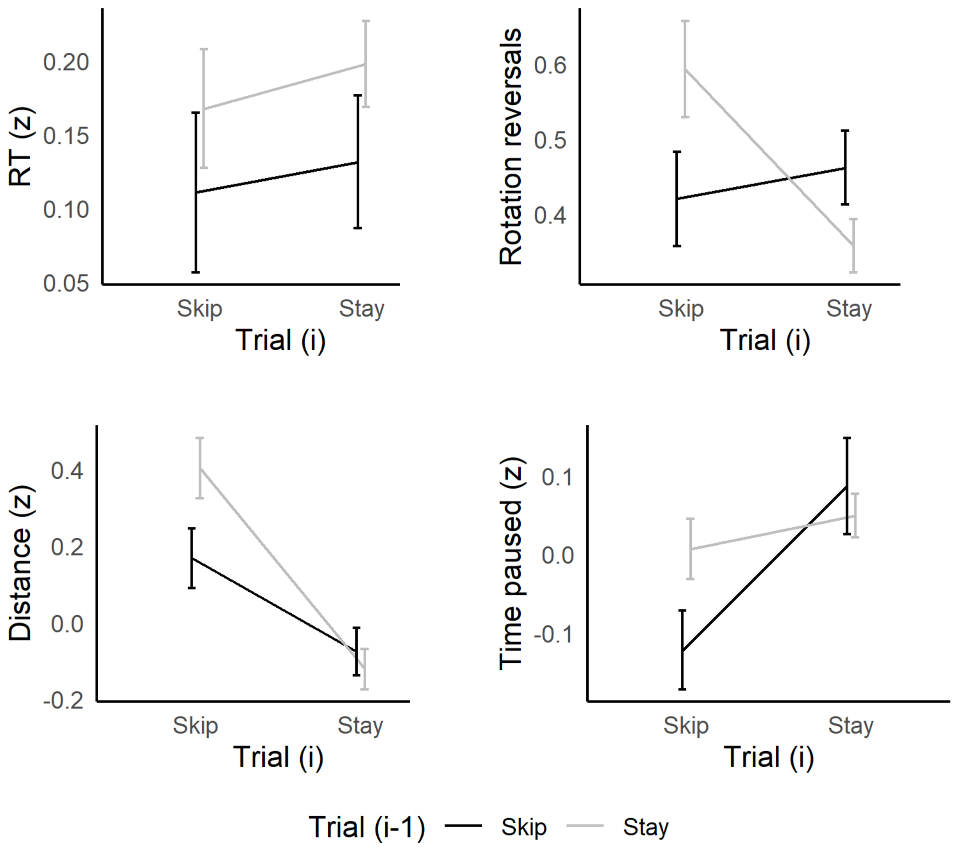

Supplementary Figure 6. Sequential choice behavior. Participants travelled farther, rotated farther and made more reversals in the direction of their rotation after previously skipping an offer (on trial *i-1*), compared to other sequences Skip/Skip sequences. These behaviors on Stay/Skip sequences were generally higher than other sequences (Stay/Stay, or Skip/Stay), except for pausing, which was more likely on average when participants accepted an offer. No significant differences were observed for reaction times.
